# Creative Destruction: A Basic Computational Model of Cortical Layer Formation

**DOI:** 10.1093/cercor/bhab003

**Published:** 2021-02-24

**Authors:** Roman Bauer, Gavin J Clowry, Marcus Kaiser

**Affiliations:** Department of Computer Science, University of Surrey, Guildford, GU2 7XH, UK; Biosciences Institute, Newcastle University, Newcastle upon Tyne NE2 4HH, UK; School of Computing, Newcastle University, Newcastle upon Tyne NE4 5TG, UK; Precision Imaging Beacon, School of Medicine, University of Nottingham, Nottingham NG7 2UH, UK; Rui Jin Hospital, Shanghai Jiao Tong University, Shanghai 200025, China

**Keywords:** agent-based model, apoptosis, cell migration, cell proliferation, cortical layer formation, neural development

## Abstract

One of the most characteristic properties of many vertebrate neural systems is the layered organization of different cell types. This cytoarchitecture exists in the cortex, the retina, the hippocampus, and many other parts of the central nervous system. The developmental mechanisms of neural layer formation have been subject to substantial experimental efforts. Here, we provide a general computational model for cortical layer formation in 3D physical space. We show that this multiscale, agent-based model, comprising two distinct stages of apoptosis, can account for the wide range of neuronal numbers encountered in different cortical areas and species. Our results demonstrate the phenotypic richness of a basic state diagram structure. Importantly, apoptosis allows for changing the thickness of one layer without automatically affecting other layers. Therefore, apoptosis increases the flexibility for evolutionary change in layer architecture. Notably, slightly changed gene regulatory dynamics recapitulate the characteristic properties observed in neurodevelopmental diseases. Overall, we propose a novel computational model using gene-type rules, exhibiting many characteristics of normal and pathological cortical development.

## Introduction

Biological tissue often exhibits complex anatomical features that are shared across many species. One well-known example is the layered distribution of different cell types seen in the mammalian neocortex, which has been a central research topic in neuroscience. A number of experimental studies have led to insights into the well-orchestrated processes that form the characteristic layers seen in the adult (Molyneaux et al. 2007; Gaspard 2011). While the pioneer of modern neuroscience Ramon y Cajal and others postulated seven layers in most neocortical regions (Kuhlenbeck 1967), Bevan Lewis (1878) mentioned the existence of six cortical layers, which was later adopted by Brodmann (1909).

Experimental work has demonstrated that the synaptic connectivity and electrical activity dynamics of cortex are strongly shaped by its layer architecture. Along those lines, the presence of connections between two cortical areas in cats and macaque monkeys can be related to the similarity of their laminar elaborations (García-Cabezas and Zikopoulos 2019; Goulas et al. 2019). Moreover, interareal connections obey generic rules dependent upon laminar identity (Markov et al. 2014). From a physiological perspective, the main prevailing model of information processing within a cortical column tightly links its functionality to the laminar architecture (Douglas et al. 1989; Douglas and Martin 2004). Although it is unknown whether layer architecture is necessary for higher order cognition, its omnipresence across different species and neural systems suggests it to be a critical factor for neural computation (Striedter 2005; Adesnik and Naka 2018). A number of experimental studies established cortical layer-specific functionalities (Bortone et al. 2014; Lur et al. 2016; Quiquempoix et al. 2018). Notably, important developments in neural imaging techniques have enabled key insights into the intricate relationship between laminar organization and electrical activity (Larkum et al. 2018).

To this day, the question of how gene regulatory dynamics give rise to the cortical neural layer architecture remains a key matter of investigation in neuroscience. Already, Wilhelm His and Ramon y Cajal studied how proliferating cells in the ventricular zone (VZ) generate neurons migrating radially toward the pia and stop before the marginal zone (MZ) (Cajal 1891; His 1904). A better understanding of how layers form during cortical development is relevant from a biological as well as medical point of view. In particular, many brain pathologies, such as in autism (Crawley 2012), schizophrenia (Nawa et al. 2000), fetal alcohol spectrum disorder (FASD) (Riley et al. 2011), or epilepsy (Bozzi et al. 2012) have been associated with developmental origins of pathological layer formation.

In the last years, in vitro models have become a viable approach to conduct research on neural development (Eiraku and Sasai 2012; Lancaster et al. 2013; Lancaster and Knoblich 2014). However, despite rapid progress along those lines, a number of serious challenges in terms of recapitulating in vivo conditions and functional behavior remain. A complementary, computational approach can benefit this research direction in multiple ways; it enables to combine various data into a coherent framework and allows to formulate and assess novel hypotheses in a quantitative manner. In particular, mechanistic computational models constitute a promising approach because they facilitate the incorporation of various genetic, molecular, and imaging data and so constitute a strong link between experiment and theory.

Only few computational/mathematical studies provide a quantitative framework for cortical layer formation. Cahalane et al. show that a mathematical model of the dynamics of neural fate determination can yield anatomical properties of superficial and deep layers of cortex across different species (Cahalane et al. 2014). However, since this framework is formulated on an abstract, analytical level, it does not take into account how factors in the physical extracellular neighborhood act on cells, such as extracellular substances and their gradients, membrane-bound proteins, or mechanical forces. Hence, it is problematic to test more direct, mechanistic hypotheses in such an approach, for example, on the involvement of certain pathways or extracellular cues. A different computational approach is taken by Caffrey et al., who employ an agent-based model-to-model cortical layer formation (Caffrey et al. 2014). This model does not comprise the developmentally crucial aspect of proliferation and relies on highly simplified intercellular interactions. Along those lines, other computational studies that model axonal and dendritic trees do not focus on the origins of a layered architecture (Koene et al. 2009; Cuntz et al. 2010). By contrast, Zubler et al. (2013) present a mechanistic computational model for cortical lamination with physical interactions. Using the “Cx3D” software (Zubler 2009), the authors studied cortical layer formation from a conceptual perspective of self-construction (Zubler et al. 2013) and put it into a gene-type coding scheme (Zubler et al. 2011; Hauri 2013). Importantly, this approach relies solely on modeling local information exchange; hence, it maintains the biologically realistic condition that cells can only experience their local 3D environment.

While the studies of Zubler et al. (2013) and Hauri (2013) propose working agent-based models for cortical layer formation, they do not demonstrate the generation of appropriate layer-specific cell numbers. Hence, it is currently unclear how a core gene regulatory network (GRN) can produce various different layer architectures. Here, we go beyond a qualitative demonstration of cortical layer formation and provide a computational model that reproduces quantitative measurements of various cortical layer architectures. In particular, we study how neocortical layer architectures observed in health and disease, across different species and cortical regions, arise. To this end, we devise a canonical GRN model, which individually plays out from a homogeneous pool of progenitor cells. In other words, we study how a core GRN, by differential alterations to its physiological interactions (Macneil and Walhout 2011), can generate a wide range of cortical layer architectures. In order to account for the evolutionary conservation of this basic structure, we reproduce experimental measurements from the human, macaque, rat, and mouse cortices. In particular, we investigate how cortical layers comprising different numbers of neurons can arise and evolve in a biologically plausible way. Importantly, this GRN model comprises the key ingredients of cortical development, that is, it incorporates the interplay between neuronal proliferation, differentiation, and apoptosis. Moreover, it respects detailed characteristics of cortical layer formation, such as an initial exponential proliferation phase that is followed by a sequential differentiation phase (Lui et al. 2011).

The overall computational model is tested in a so-called agent-based approach: initially, a homogeneous pool of neuronal precursors is represented, where each cell’s behavior is guided by its individual GRN model as predicted by the protomap hypothesis (Rakic et al. 2009). This model constitutes the genetic rules that specify the cellular dynamics of the individual cells (hence, each cell acts as an autonomous “agent”). Based on this initial configuration, simulations are conducted to demonstrate how, from the proliferating cell population, the genetically specified dynamics play out and yield laminar neuron numbers in accordance with experimental observations.

## Materials and Methods

### Layer-Specific Neuron Numbers

Experimentally obtained measurements of layer-and species-specific neuron numbers were obtained from (O’Kusky and Colonnier 1982; Schüz and Palm 1989; DeFelipe et al. 2002). Layer thicknesses from human cortex were obtained from von Economo (2009). These data were used to assess the impact of genetic alterations in neurodevelopmental disorders (Fig. 7, Supplementary Figs 3 and 4) and to analyze the correlations between layers (Supplementary Fig. 2).

### Images

The histology sections shown in Figure 7 were obtained from Casanova (2007) and Golden and Harding (2010).

### Simulation Environment

The simulations were conducted using the agent-based open-source framework “Cx3Dp,” the parallelized version of Cx3D (Zubler 2009; available at http://www.ini.uzh.ch/projects/cx3d/).

Our computational model includes a GRN that is instantiated in a few precursor cells at the beginning of the simulation. In our model, each of the cells follows internal, gene-type rules, while physically interacting with the local, extracellular environment. The GRN implementation is based upon the Cx3D LocalBiologyModule class, where each module defines specific cellular behaviors. The symmetric division phase is governed by a dedicated module, incorporating recursive cell division and growth. Upon termination of symmetric division, this module invokes the differentiation module, which can also probabilistically invoke A1-type apoptosis. After differentiation has occurred, the migration module is initiated, which finally invokes the A2-type apoptosis module. Mechanical forces also act between neighboring cells that are in physical contact. These cell–cell interaction forces are computed based on the diameter of spherical somata, their relative distance, and their adhesive properties. Moreover, cells can secrete chemicals that diffuse extracellularly in 3D space in a gradient fashion and that can be sensed by other cells. For more information on the framework, please refer to the relevant Supplementary Material Section 1 and Zubler (2009). Notably, our GRN implementation is independent from the GRNs described in Hauri (2013), Zubler et al. (2013).

### Usage of Experimental Data

In order to compare layer-specific neuron numbers with the computer simulation results, the number of neurons per layer under a cortical surface area of 300 μm × 300 μm was computed. Moreover, since these numbers reflect the neuronal statistics in the adult cortex, the number of neurons dying postnatally (which was not included in our study), estimated to amount to approximately 25% of all neurons (Heck et al. 2008), was added to the adult-stage neuron numbers. Hence, the numbers listed in Table 1 are multiplied by $0.3\cdot 0.3\!\Big/ \!0.75\Big.$. Layer thicknesses used to inform the phenomenological model were obtained from von Economo (2009). Time stamps in Figure 3 are based on Saito et al. (2011), Workman et al. (2013).

**Table 1:** Estimated layer-specific numbers of neurons across different species, based on data from DeFelipe et al. (2002) for human, rat, and mouse cortices and from O’Kusky and Colonnier (1982) for macaque cortex

# of neurons under 1 mm^2^ in	Human temporal cortex	Rat hindlimb somatosensory cortex	Mouse barrel cortex	Macaque visual cortex
Layer 1	1958	427	1258	600
Layer 2/3	27 514	28 183	32 347	56 100
Layer 4	13 158	13 827	37 723	90 300
Layer 5	12 738	20 785	19 286	24 800
Layer 6	8052	36 322	55 063	30 100

To estimate the biological time spans for the developmental phases of the model, the start and end times were determined based on the study of Workman et al. (2013). In particular, the progenitor amplification phase was estimated to start at embryonic day 30 in humans (Bystron et al. 2008). Associated times in rat, mouse, and macaque monkey were estimated based on the work of Workman et al. (2013). The selected start and end points for the differentiation phase were determined based on the “Neurogenesis cortical layer VI start” and “Neurogenesis cortical layer II/III end” events, respectively.

### Simulation Setting

Initially, 175 precursor cells are arranged as a 2D layer comprising an area of 300 μm × 300 μm, where the cell body locations are given by (normally distributed) random displacements from regular grid points with a jitter of 0.3 μm standard deviation. The simulation takes place within a 3D environment with columnar boundaries of size 300 μm × 300 μm × 1400 μm, the latter being the radial dimension.

Standard Cx3Dp cell body parameters are chosen for these. Each precursor cell is initialized with an intracellular substance quantity of 100 (unitless), which decays by 1% at each time step. The end of the initial, symmetric proliferation phase is invoked when this substance quantity falls below a prespecified threshold *T*_SYM_. 50 000 times steps were simulated to generate the final laminar arrangement. The model produced systems containing approximately 7000, 11 000, 16 000, and 24 000 cell bodies in human, rat, mouse, and macaque cortex simulations, respectively.

**Table 2:** Parameters of GRN model for layer formation

**Commitment probabilities**	**C**_**M**_	**C**_**6**_	**C**_**5**_	**C**_**4**_	**C**_**3**_	**C**_**2**_
Human temporal cortex	0.16	0.1	0.2	0.3	0.5	0.7
Rat hindlimb somatosensory cortex	0.08	0.2	0.2	0.3	0.5	0.6
Mouse barrel cortex	0.14	0.3	0.2	0.5	0.5	0.4
Macaque visual cortex	0.11	0.2	0.2	0.5	0.4	0.5
**Apoptosis probabilities and Timing threshold**	**P**_**M**_	**P**_**6**_	**P**_**5**_	**P**_**4**_	**P**_**3**_	
Human temporal cortex	0.7	0.65	0.55	0.5	0.3	
Rat hindlimb somatosensory cortex	0.55	0.6	0.7	0.5	0.5	
Mouse barrel cortex	0.45	0.6	0.5	0.4	0.5	
Macaque visual cortex	0.6	0.4	0.2	0.5	0.5	

Notes: The parameters are listed, specifying the probabilities to commit to a specific neuron type (*C_M_*–*C*_2_, for MZ/layer 1 to layer 2) and the apoptotic probabilities at each stage (*P_M_*–*P*_3_, see Fig. 1). The final apoptotic probability *P*_2_ fulfills the criterion *P*_2_ + *C*_2_ = 1 and so is not listed as a model parameter

### Model Parameters

The maximum cell body diameter during proliferation and differentiation was set to 10 μm. If this value is reached during cell body growth, the cell body either divides and subsequently increases its diameter during growth (the diameter growth per time step was 0.2 μm), or terminates its differentiation pathway. However, after a cell has terminated migration and has taken up its final location within the layer architecture, it grows its diameter to 15 μm.

Certain physical parameters change dynamically during the simulation in order to improve the segregation between the individual layers. In particular, MZ cell bodies, that have reached their prespecified external substance concentration and stopped their migratory mode, adopt the Cx3D parameters “interobject-force” coefficient of 1, “adherence” of 0.0001, and a “mass” value of 0.0001. The (standard) interobject-force coefficient of 1.0 renders the MZ cells responsive to mechanical forces from migrating cells. The small adherence and mass values enable them to be easily pushed, for if their mass were large, they would remain at their original position. In other words, these model parameters allow for the migratory cells to push the MZ cells along the radial direction. If not stated otherwise, all other cells had the standard Cx3D values for these three model parameters, that is, 1.

The GRN model of differentiation has 12 parameters. These comprise six probabilities to commit to layer-specific neuron types (*C_M_*, *C*_2_, *C*_3_, *C*_4_, *C*_5_, and *C*_6_). Moreover, the six parameters *P_M_*, *P*_2_, *P*_3_, *P*_4_, *P*_5_, and *P*_6_ determine the probability to commit to apoptosis of type A1. For simplicity, *C*_2_ + *P*_2_ = 1 was used, so only 11 of the 12 parameters were varied to match the experimental data. Parameters were determined in a semi-heuristic process: All parameters were initially set to 0.5. In accordance with the temporal order of layer formation, associated commitment and apoptosis parameters were changed sequentially in order to generate appropriate neuron numbers. The mathematical analysis described in the Results section was used to guide this parameter selection process. The (fixed) parameter *T*_SYM_ determines the number of symmetric cell divisions in the early proliferation phase and is set identical for all the layer architectures here due to lack of empiricaldata.

MZ cells that have terminated migration each create a physical bond with a neighboring cell, with the Cx3D physical bond parameters “breaking-point” set to 500%, the “dumping constant” set to 0, and the “spring constant” set to 1. This physical bonding enables MZ cells to remain connected with one another. By contrast, cell bodies of the other layers (2–6) assume a mass of 1 during migration and increase their mass to 10 after the termination of migration. Moreover, migratory cells’ interobject-force coefficients assume a value of 0.0001. Overall, these physical parameter settings facilitate the right developmental operation: Heavy cells push the lighter MZ cells before taking up their final position. Preliminary simulations indicate that limited variations of these parameters do not significantly alter the resulting cell distributions.

The stopping of cell migration is induced when certain conditions are fulfilled. In the case of layers 2, 3, 4, and 5 cells, two conditions need to be fulfilled: The number of MZ cells in the local neighborhood, that is, in the physical space where physical interactions can take place, must exceed one. Moreover, the number of neighboring cells of the other, earlier developing layers (e.g., in the case of layer 4 cells, these would be layers 5 and 6) must be zero. In the case of layer 6 cells, at least one MZ cell must be detected. Finally, in the case of MZ cells, a prespecified extracellular cue concentration needs to be reached. To this end, an extracellular substance gradient that increases in concentration toward the pia, is instantiated when simulations start. These conditions enabled cells to recognize their appropriate locations and establish the associated layer thicknesses, while pushing the MZ toward thepia.

Once cells have stopped, they initiate their apoptosis modules that trigger cell death if a given condition is fulfilled. Neurons belonging to a specific layer sense the number of local, neighboring neurons (distance <20 μm) belonging to a different layer. If at least three neurons belong to neighboring layers, or one belongs to a more distant layer (e.g., layer 6 for neurons in layer 3), this condition is fulfilled, and (A2-type) apoptosis is triggered. Since the number of layer 1 cells is very low in all species, an additional probabilistic apoptotic rate was included only for layer 1 cells, which does not depend on extracellular conditions. This apoptotic probability was set to *P* = 0.9 for all species, which yielded agreement with the experimentally measured neuron numbers in layer 1.

The parameters for the GRN are shown in Table 2. They were manually adjusted by taking into account that increased apoptosis at a given stage reduces the number of subsequently developing neurons and increased commitment of a proliferative state to a given neuronal layer type decreases the number of neuronsleft.

### Analysis of Layer Properties

After simulations terminated, the cell positions and information on their cell identity (i.e., which layers they belong to) were exported as a matrix into a Matlab-readable file. Since (occasionally) cells migrated through the MZ and ended up at the topmost radial position, these were discarded in the analysis by thresholding.

The histograms shown in Figures 4 and 7*E*,*F* were computed using Matlab. In order to better account for the variation of layer-specific cell numbers, the number of bins were adapted for improved visibility and accuracy. Layer-specific neuron numbers that are compared in Figure 5 were also computed using Matlab. Agreement between experimental and simulated neuron numbers was determined if the mean simulated number was within the 95% confidence interval of the experimentally measured distribution.

## Results

### The Model

**Figure 1:**
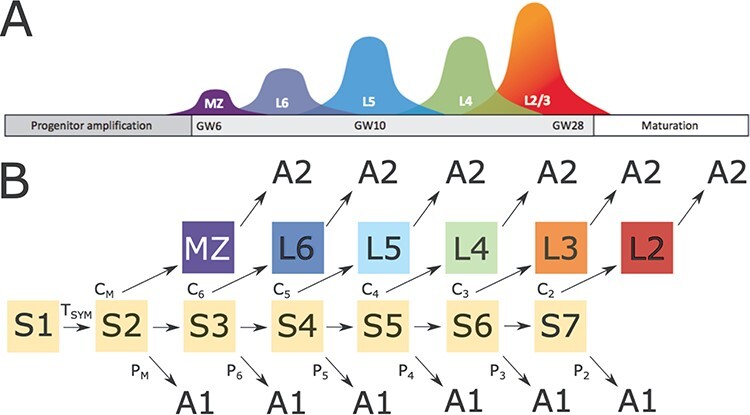
(*A*) Schematic of sequence of cortical layer formation. During cortical development, progenitor cells proliferate and differentiate into different neuron types in a consistent sequence. MZ cells (dark blue) are the earliest cells to be produced. Afterward, differentiation into layer 6 (blue), layer 5 (cyan), layer 4 (green), and layer 2/3 (purple) cells occurs. (*B*) Structure of state diagram of cortical lamination model. Arrows indicate (probabilistic) paths of the network constituting the state diagram. The initial, purely proliferative state S1 leads to state S2 when an internal timer triggers this state transition. From this second proliferative state S2, neuronal migration of MZ cells is triggered with probability *C_M_* (the MZ is the predecessor of layer 1). Alternatively, apoptosis (A1-type) is invoked, or the cell divides again to proceed to the next neural progenitor state S3. S3 cells are the progenitors of layer 6, layer 5, layer 4, or layer 2/3 cells. During this sequential differentiation process, apoptosis occurs with probabilities *P_M_* to *P*_2_. At the end of the lamination process, a final apoptotic step (A2) occurs that depends on the local neighborhood of cells. *T*_SYM_ denotes a threshold for an internal clock and determines when the symmetric division phase stops. Yellow color indicates stem cell states, that is, these cells have the potential to divide. Dark blue, light blue, cyan, green, orange, and red indicate final cell types.

We studied two phases during cortical development, that is, the progenitor amplification phase, when the neural progenitor cell pool increases exponentially due to recursive cell division. In the second phase also differentiation and migration of neurons is modeled. Figure 1*A* visualizes these phases.

#### The Progenitor Amplification Phase

The simulation starts with the progenitor cells that are arranged randomly on a 2D sheet, which represents the proliferative zone. Each of these cells follows their own gene regulatory dynamics, specifying recursive cell growth and division (Fig. 1*B*). The initial state of the GRN is S1. In this first stage of proliferation, cells divide and grow recursively, producing an exponentially increasing number of neural progenitor cells (S2) (Lui et al. 2011). Squares colored yellow indicate cell states with proliferation capacity.

In our model, the proliferating cells possess an internal clock that is realized by means of an intracellular substance. This substance decays over time, and once the progenitor cells sense that it is below a prespecified threshold T0, they terminate this first proliferative stage of symmetric division. These progenitor cells then transition to stage S2, which is the first GRN state that leads to neuronal differentiation. Hence, this intracellular substance acts as a trigger for the neuron specification phase.

In terms of biological time, in human, rat, mouse, and macaque, cortical development the progenitor amplification process starts approximately at 30 days, 9.9 days, 8.4 days, and 34 days postconception, respectively.

**Figure 2:**
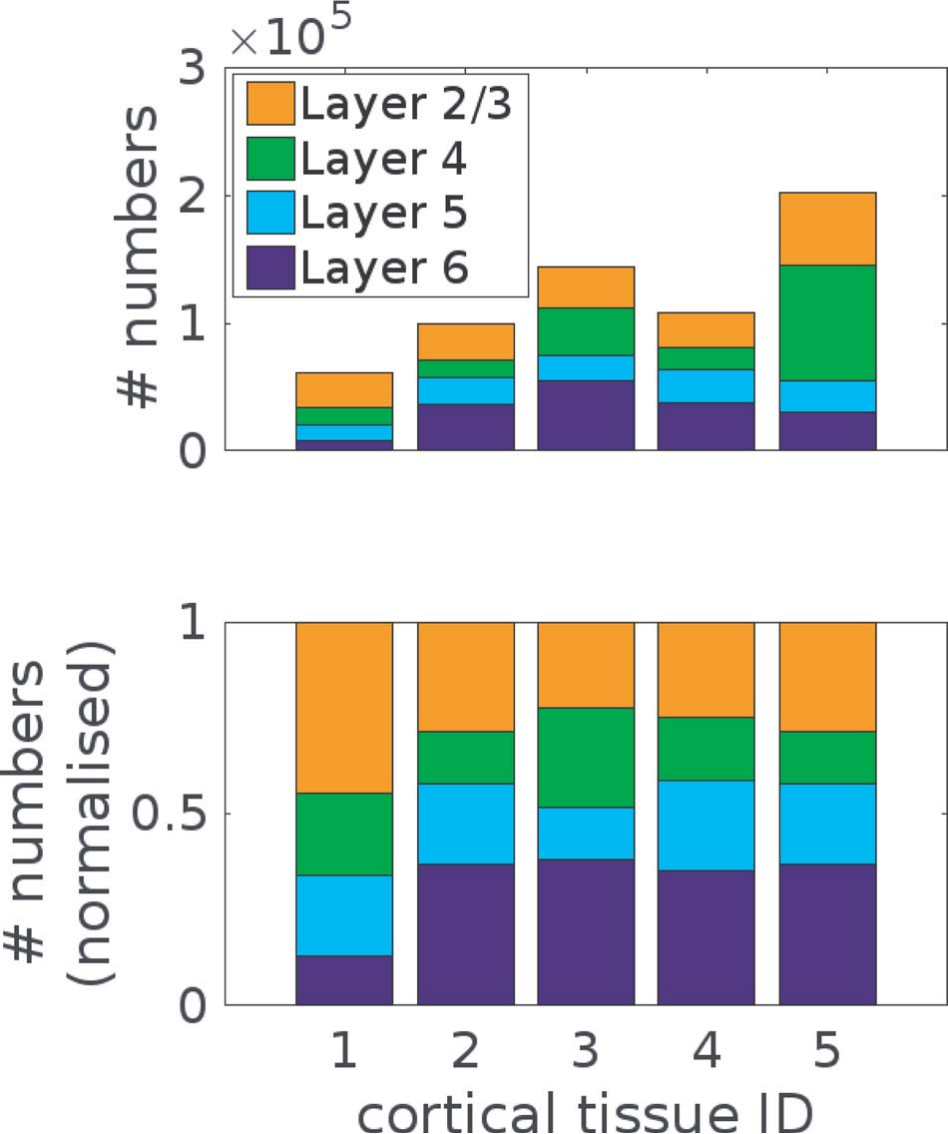
Experimentally observed number of neurons under 1 mm^2^ of cortical surface area, in the cortical layers of different areas and species. The shown examples are: human temporal cortex, rat somatosensory cortex, mouse barrel cortex, mouse frontal cortex, and macaque visual cortex. There is substantial variation in the number of neurons across individual layers (*A*) as well as the relative proportions (*B*) within the same cortical area and species.

#### The Differentiation Phase

After the exponential proliferation phase (instantiated within the cell state S1), the progenitor cells still retain the potential to proliferate, but they additionally can probabilistically commit to various other cell types (Gao et al. 2014). Once this differentiation process has occurred, neurons migrate radially to take up their final location within the cortex.

Initially, the neuronal progenitor cells (probabilistically) either differentiate into MZ cells, progress further along the differentiation path by dividing, or undergo apoptosis. States S2–S7 constitute proliferative stem cell states, where (for simplicity) only one round of proliferation can occur.

The MZ develops by MZ cells migrating along the gradient of a predefined extracellular cue that increases in concentration along the radial direction. The MZ cells continuously sense this chemical cue, and once a given threshold is reached, they stop migrating. The layer structure is not influenced by the magnitude of this threshold, which solely determines the distance between the proliferative zone and the developing cortex. It mimics the influence of the pial membrane, which in reality stops migration of the MZ cells.

For simplification, we model that layer 6 migrates separately from MZ cells, although in reality layer 6 splits the so-called preplate into MZ and subplate. In our model, layer 6 cells also commit to migration after a brief waiting period (that is specified again based on a decaying intracellular substance acting as a cellular clock). Because of the waiting period, L6 cells migration occurs after MZ cells have reached their target, and L6 cells stop migrating as soon as they sense MZ cells.

The rest of the differentiation process is a repetition of this elemental process of differentiation, proliferation, and apoptosis: Those precursor cells that did not commit to the MZ or L6 fate will continue to divide. After dividing, they again probabilistically commit to the neuronal fate (i.e., layer 5), continue with proliferation, or undergo apoptosis, and so on. This developmental process can be very efficiently encoded in the GRN because it is composed of repetitions of the same elemental geneticrule.

The parameters of the GRN are given by T1, which is a threshold that determines when the symmetric division phase ends. As soon as a cell-internal substance decays below T1, the progenitor amplification phase ends. In other words, T1 determines the number of cells before sequential differentiation is induced. In all simulations, T1 is set to 65. The probabilities *C_M_*, *C*_6_, *C*_5_, *C*_4_, *C*_3_, and *C*_2_ determine the probabilities for progenitor cells to commit to differentiated neuron types; *P_M_*, *P*_6_, *P*_5_, *P*_4_, *P*_3_, and *P*_2_ are the probabilities for progenitor cells to commit to apoptosis. To demonstrate the capabilities of the model, these parameters were adapted to produce the experimentally measured thicknesses.

**Figure 3:**
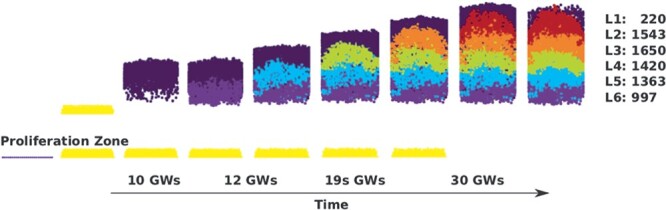
Sequence of lamination steps. In this simulation of layer formation in the peristriate area of the human temporal cortex, initially only a small set of progenitor cells exist in the VZ (left, dark blue). This proliferative zone (yellow) generates neuronal precursors in an exponential manner. These precursors, via symmetric and asymmetric division, give rise to different cortical layers. The first cells that migrate are MZ cells (dark blue, top). Subsequently, layer 6 cells (blue) physically push the MZ, constituting a mechanistic process that is repeated for the layers 5 (cyan), 4 (green), 3 (orange), and 2 (red). Hence, the layer architecture is produced in the characteristic outside-in manner of cortical layer formation. In humans, the overall formation of cerebral cortex happens approximately between gestational weeks 8 and 30.

In terms of biological time, in human, rat, mouse, and macaque cortical development, the differentiation process starts approximately at 48 days, 12 days, 11 days, and 45 days postconception, respectively. It ends at about 191 days, 23 days, 22 days, and 112 days postconception, respectively.

### Consistency of GRN Structure

One remarkable observation across different cortical areas in humans as well as in other species is the significant variation of cell numbers in individual layers (Fig. 2; Herculano-Houzel et al. 2008; Collins 2011). It follows that an important condition for a mechanistic generic computational model of layer formation is the possibility to account for various compositions of layer-dependent cell numbers.

**Figure 4:**
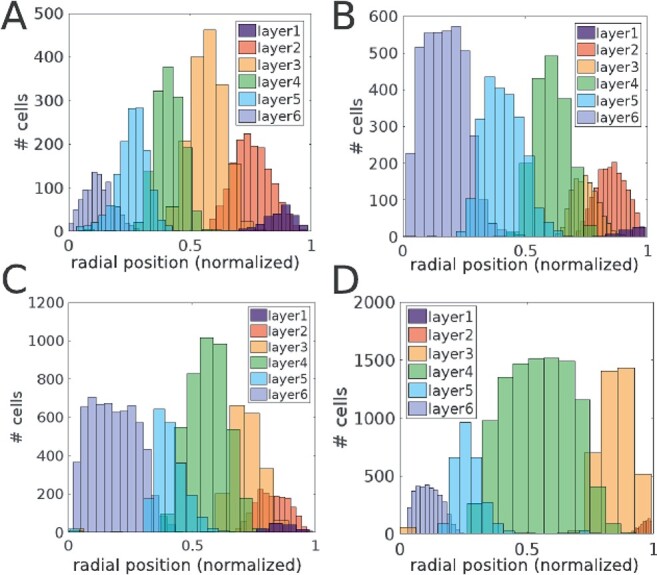
Spatial distributions of (computationally simulated) layer-specific neuronal locations along the radial axis. The histograms demonstrate that cell types, as defined by their “genetic” state, are indeed well segregated along the radial direction, comprising distinct layers with different cell types. The shown layer architectures are from (*A*) human temporal cortex, (*B*) rat somatosensory cortex, (*C*) mouse barrel cortex, and (*D*) macaque visual cortex. Different colors indicate different layers.

We first demonstrate that our model can yield a wide range of neuronal numbers across layers within different cortical areas and species. Figure 3 shows the sequential development of the layers in the human temporal cortex in the correct order, that is, the characteristic inside-out dynamics of layer formation is recapitulated (see also Supplementary Video 1). Moreover, the development of the layers in rat, mouse, and macaque cortices was simulated again by introducing changes to the model parameters. The resulting distributions of neurons across different layers are shown in Figure 4.

**Figure 5:**
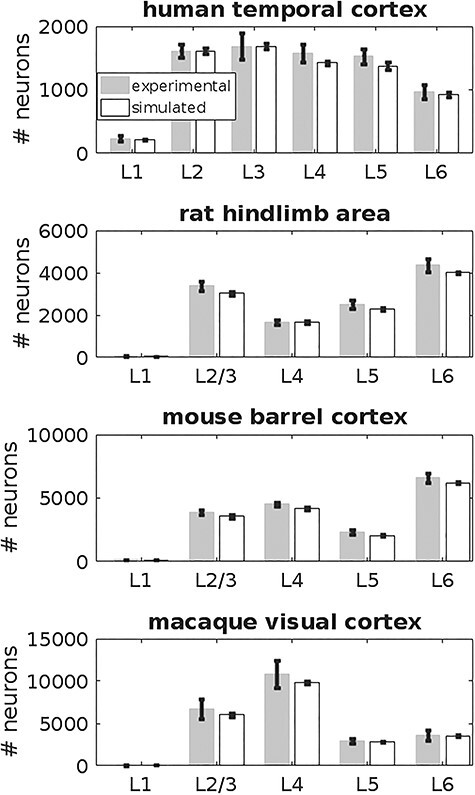
Layer-specific neuron numbers in human, rat, mouse, and macaque cortical tissue, under 300 μm × 300 μm of cortical surface. Numbers based on experimental measurements and based on computer simulations are shown in blue and green, respectively. The simulated layer architectures are in accordance with the experimentally obtained measurements, demonstrating that the model can consistently account for a wide range of neuron numbers. Error bars indicate standard deviation as obtained from experimental data and from 10 simulations, respectively. The null hypothesis that the means of the simulated layer-specific neuron numbers stem from the experimentally derived numbers could not be rejected based on a two-sided *t*-test with significance level α = 0.05, for all the showndata.

In Figure 5, the experimental data obtained from multiple experimental studies are shown together with the corresponding simulated layer dimensions, highlighting the explanatory power of the simple GRN structure shown in Figure 1*B*. Overall, these results demonstrate that inter-species variation of layer-specific cell numbers can be accounted for only by modifications to the parameters of the GRN rather than structural changes.

**Figure 6:**
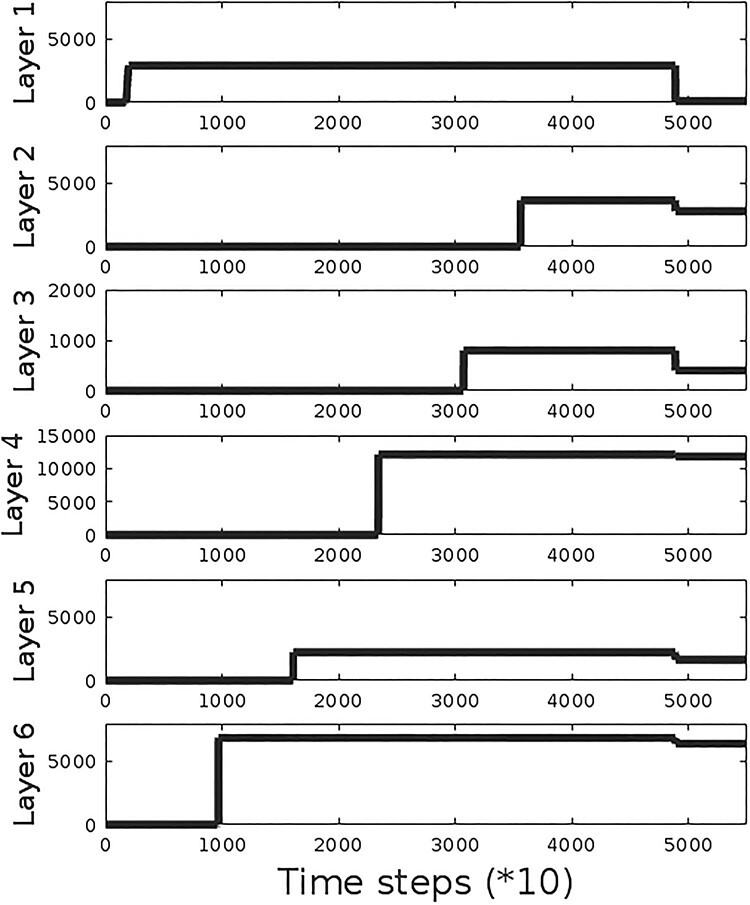
Neuron numbers in individual layers during layer formation. The GRN defines a sequential differentiation process, whereby the individual layers originate in a predefined temporal order from the proliferative zone. This developmental sequence expresses itself by a rise in neuron numbers, whereby first the MZ (later constituting layer 1) develops. Subsequently, the deep layers form before the superficial layers. Finally, neurons that recognize their own mislocation by identifying their immediate neighbor neuron types enter the apoptotic state A2, which yields a decline in neuron numbers. In our model, this apoptotic state is triggered by layer 2 cells that occupy their terminal location after migration and signal this event to the other cells via an extracellularly diffusing substance. Note the large proportion of apoptosis for layer 1 cells, which stems from the fact that these cells are pushed by the later-developing neurons and so exhibit a higher risk for mislocation.

Figure 6 shows the cell number dynamics in a simulation of macaque cortical layer formation. First, the MZ that will subsequently become layer 1 develops and then layers 6, 5, 4, 3, and 2 form. The initial production from the stem cell pool is followed by reductions in cell numbers through the apoptotic stage A2 (Fig. 1). We find that most of the apoptosis occurs in mislocated MZ cells, predominantly in the superficial cortical layers.

The modifications of the GRN are solely changes in probabilities for transitions between certain states rather than changes to the GRN states or the connections themselves. This general applicability of the GRN structure indicates that minor evolutionary changes can yield a wide range of phenotypic differences in brain structure. In our model, these modifications can include changes to apoptotic rates in addition to changes to cellular fate commitment. However, these model parameters are underconstrained by current experimental data; hence, there are different possibilities of GRN parameter sets yielding equivalent layer-specific cell numbers. Supplementary Figure 1 shows how changes in exemplary parameters of the GRN affect the resulting layer-specific neuron numbers: Depending on where in the GRN the given model parameter takes effect, the subsequently developing layers are affected.

### Differential Apoptotic Processes

In our model, changes to apoptosis at early stages affect the final cell numbers. Apoptosis later during development has less impact on the total tissue thickness because only later-developing layers will be affected. Nevertheless, all the probabilities for cells in the proliferative stages to exit and commit to apoptosis (GRN states labeled A1 in Fig. 1*B*) exert control over cell numbers. Overall, changes to these probabilities can yield extensive differences in the final cell numbers and layer dimensions and so the A1-type apoptotic stage is paramount in generating the right number of cells.

In a second process, apoptosis is a crucial determinant of the proper layer architecture and neuronal density (GRN states labeled A2 in Fig. 1). Our model of this second type of apoptosis is inspired by Hauri (2013): Cells can sense their local environment, and based on the neighboring cell types, commit to apoptosis. Whenever a cell senses too many neurons of a type that is different to its own, it commits to apoptosis. Hence, in this second phase of apoptosis, a different pathway is activated because it involves the binding of extracellular ligands (Guerin et al. 2002).

In our model, this A2-type apoptosis is initiated after the end of migration when the first layer 2 cells have reached their final radial position and then trigger the apoptotic process. To this end, these apoptosis-initiating layer 2 cells secrete a diffusible substance, which is then detected by all cells that have reached their final destination. Once a sufficient amount of this factor is detected in their neighborhood, these cells sense the number of cells different from their own type. If this number exceeds a prespecified threshold, the cell commits to apoptosis. This type of apoptosis that happens after migration in our model is crucial for refining the layered architecture of the final cortical structure.

These simulation results point toward apoptosis as a highly heterogeneous mechanism. In the first apoptotic stage, the fate is determined probabilistically according to a prespecified chance that is not influenced by external factors. In the second apoptotic stage, extracellular cues conveying information on the identities of neighboring cells are taken into account. In our simulations, we observe that this apoptotic behavior is particularly important for layer 1, which during development is successively pushed by the deeper layers; it occurs more often for this layer that some cells are not pushed effectively and hence remain stuck in a deeper position than the target layer.

### Mathematical Analysis of Layer-specific Neuron Numbers

In our model, the number of neurons per layer is determined by overall 11 GRN parameters. These parameters specify the probabilities of either committing to a certain layer neuron type, or continuing proliferation, and then potentially committing apoptosis. These parameters are *C_M_*, *C*_6_, *C*_5_, *C*_4_, *C*_3_, *C*_2_, *P_M_*, *P*_6_, *P*_5_, *P*_4_, and *P*_3_ (*P*_2_ is given by 1 − *C*_2_). Based on these parameters, we derived the number of neurons per layer (the GRN is inherently stochastic and so we estimate the expected number of neurons).

We first indicate the number of undifferentiated precursor cells at state S2, after the symmetric division phase has ended, by *N_P_*. The number of layer 1 neurons is proportional to *N_P_* and the probability to commit to the MZ cell fate *C_M_*:

$$ {N}_1\approx 2{N}_P{C}_M.$$

The factor 2 stems from the proliferative state S2 (Fig. 1*B*), where cells undergo a round of cell division (state S2 in Fig. 1*B*). ${N}_1$ cannot be computed exactly because of the inherent stochasticity of the GRN dynamics and the impact of type-2 apoptosis (A2). The number of layer 6 neurons can be estimated as:

$$ {N}_6\approx{2}^2{N}_P\left(1-{C}_M\left)\right(1-{P}_6\right){C}_6.$$

Again, the factor 4 (=2^2^) stems from the proliferative cell state (state S3 in Fig. 1*B*). Since only precursor cells that neither commit to layer 1 nor to apoptosis, the factors (1 − *C_M_*) and (1 − *P_M_*) are included. Moreover, the term is multiplied by *C*_6_ as the probability to commit to layer 6.

Analogously, the remaining neuron type number estimates can be computed as:

$$ {N}_5\approx{2}^3{N}_P\left(1-{C}_M\right)\left(1-{P}_M\right)\left(1-{C}_6\right)\left(1-{P}_6\right){C}_5,$$

$$ {N}_4\approx{2}^4{N}_P\left(1-{C}_M\right)\left(1-{P}_M\right)\left(1-{C}_6\right)\left(1-{P}_6\right)\left(1-{C}_5\right)\left(1-{P}_5\right){C}_4,$$

$$ {N}_3\approx{2}^5{N}_P\left(1-{C}_M\right)\left(1-{P}_M\right)\left(1-{C}_6\right)\left(1-{P}_6\right)\left(1-{C}_5\right)\left(1-{P}_5\right)$$

$$ \left(1-{C}_4\right)\left(1-{P}_4\right){C}_3,$$

$$ {N}_2\approx{2}^6{N}_P\left(1-{C}_M\right)\left(1-{P}_M\right)\left(1-{C}_6\right)\left(1-{P}_6\right)\left(1-{C}_5\right)\left(1-{P}_5\right)$$

$$\left(1-{C}_4\right)\left(1-{P}_4\right)\left(1-{C}_3\right)\left(1-{P}_3\right){C}_2.$$

To show how apoptosis increases the flexibility of the layer-specific neuron numbers, let us take *N*_3_ as an example. *N*_3_ is dependent upon parameters of differentiation and apoptosis, that is, the probabilities for cell fate commitment *C_M_*, *C*_6_, *C*_5_, *C*_4,_ and *C*_3_ as well as apoptosis probabilities *P_M_*, *P*_6_, *P*_5_, and *P*_4_. Hence, if there was no apoptosis, changes in any previously developing layers (e.g., layer 5) would necessarily entail changes also in the later-developing layers. In our proposed GRN model, it is possible for *N*_5_ to change without affecting *N*_4_, *N*_3_, and *N*_2_ because apoptosis and cell fate commitment rates can balance out previous changes. Hence, the inclusion of apoptosis enables layers to develop individually, allowing for a high adaptivity of the layer architecture. From an evolutionary perspective, this enables layer architectures to adapt to different requirements with regard to their inputs, outputs, and intra-areal circuitry.

To further highlight the benefit of apoptosis, the equations for the above model without apoptosis would be:

$$ {N}_6\approx{2}^2{N}_P\left(1-{C}_M\right){C}_6,$$

$$ {N}_5\approx{2}^3{N}_P\left(1-{C}_M\right)\left(1-{C}_6\right){C}_5,$$

$$ {N}_4\approx{2}^4{N}_P\left(1-{C}_M\right)\left(1-{C}_6\right)\left(1-{C}_5\right){C}_4,$$

$$ {N}_3\approx{2}^5{N}_P\left(1-{C}_M\right)\left(1-{C}_6\right)\left(1-{C}_5\right)\left(1-{C}_4\right){C}_3,$$

$$ {N}_2\approx{2}^6{N}_P\left(1-{C}_M\right)\left(1-{C}_6\right)\left(1-{C}_5\right)\left(1-{C}_4\right)\left(1-{C}_3\right){C}_2.$$

In contrast to the model with apoptosis, here there is a strict dependency between individual layers. For instance, the number of layer 3 neurons decreases if the number of layer 5 neurons increases. There is no evidence for such consistent relationships between individual layers. Along those lines, Supplementary Figure 2 shows an analysis of layer thickness correlations in the human brain (von Economo 2009), without a single instance where layer thicknesses were negatively correlated. Hence, we propose that apoptosis in cortical development enables cortical layers to change individually.

### Layer Architecture in Neurodevelopmental Disorders

**Figure 7:**
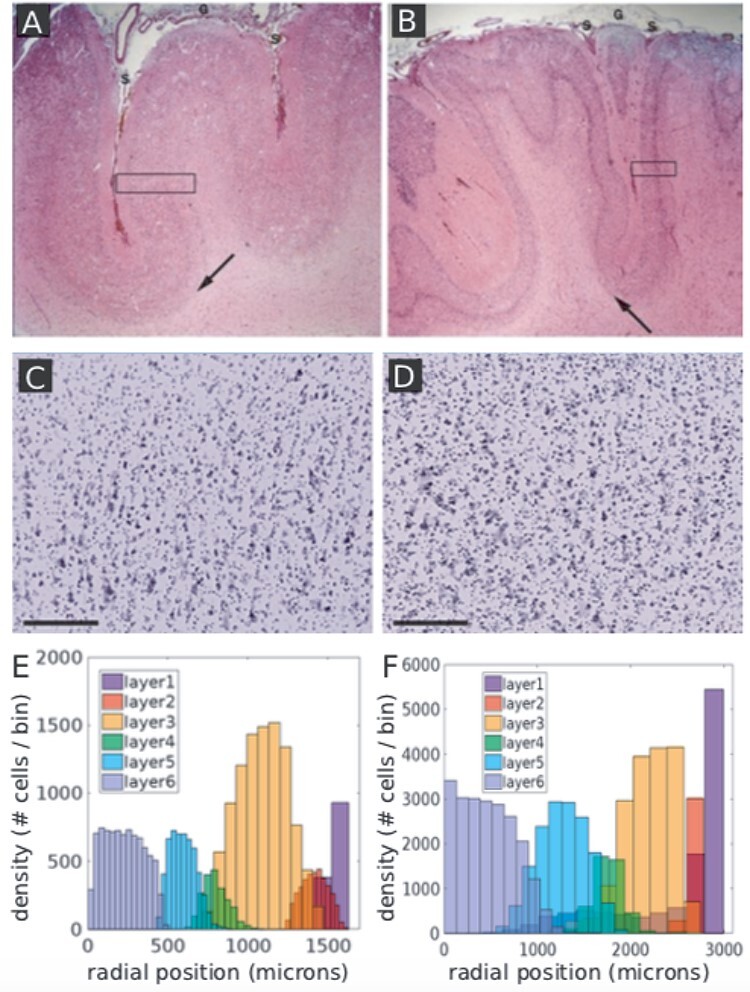
Pathological properties in polymicrogyria and autism can be recapitulated using a phenomenological model of cortical layer formation. (*A*) Histology section from healthy human cortex and a human cortex with polymicrogyria (*B*). Patients with polymicrogyria often have a reduced cortical thickness, while more and smaller gyri can be observed. The length of the boxes indicates the different cortical thicknesses. In both scenarios, the layer structure is usually preserved and the boundary between cortex and white matter is well defined (arrows). (*C*, *D*) Nissl-stain from healthy (*C*) and autistic (*D*) cortex (cortical area 9, right hemisphere, layer 3). Experimental observations in the autistic cortex show a higher cell density and more jittered cell arrangement. Scale bars measure 200 μm. (*E*, *F*) Intriguingly, we could recapitulate thinner cortices or increased cell density by a single parameter change. (*E*) Increased (A1-type) apoptosis during early proliferation and differentiation yields simulation results resembling a pathological phenotype, where the cortical thickness is approximately 1.5 mm instead of the control simulation (2.7 mm in human peristriate area, Supplementary Fig. 3*A*). (*F*) Dysfunctional apoptosis (A2-type, as indicated in Fig. 1*B*) yields higher cell densities and more mixed positions, reminiscent of observations from the autistic cortex. Especially layer 1 neurons are affected because they are pushed during the entire layer formation process, yielding many cells that are not pushed correctly to the correct position along the radial axis. Without A2-type apoptosis, there is significantly less segregation between neighboring layers as indicted by the overlap of the histograms. Moreover, the cortical thickness is slightly increased (3.0 mm instead of 2.7 mm).

In addition to layer architectures in healthy cortex, we also investigated whether our model could capture characteristics of certain neurodevelopmental disorders (Fig. 7*A*–*D*). Evidently, there is a large variation of the phenotypic expression across pathologies. Nevertheless, specific general properties of malformation and developmental causes are consistent within types of disorders (Barkovich et al. 2005). To investigate whether our model can generate characteristics reminiscent of certain neurodevelopmental disorders, we conducted small changes to the probability parameters of our computational model. To this end, we studied the impact on cortical thickness, which has been experimentally assessed in certain diseases (in contrast to the number of neurons). As the control, we simulated based on our GRN model a layer structure that is in agreement with the layer thicknesses of the human peristriate area 19 (Supplementary Fig. 3*A*). The cortical thickness of this specific area has been measured to be approximately 2.7 mm (von Economo 2009).

### Polymicrogyria

Polymicrogyria is a neurodevelopmental disorder that usually entails a reduced cortical thickness and an increase in folding patterns, while a clearly layered structure is still preserved (Golden and Harding 2010; Judkins et al. 2011). We assessed the phenotypic impact of increased A1-type apoptosis, that is, the scenario where apoptotic activity at early developmental stages (A1-type) was significantly increased. As shown in Figure 7*E*, these simulations recapitulate a reduced cortical thickness (~1.6 mm) compared with the control thickness (~2.7 mm, see Supplementary Fig. 3*A*), while laminar organization is still preserved.

In addition to the scenario where apoptosis at early stages was increased, we conducted simulations where the initial phase of proliferation stopped prematurely. This decreased the neural progenitor pool significantly but did not interfere with the migration and apoptosis (Supplementary Fig. 3*B*). Again, a reduced cortical thickness was observed also in this scenario, while the layer thicknesses were proportionally preserved. Hence, genetic changes reducing the proliferation in the exponential proliferation stage, or increasing A1-type apoptosis, can lead to the phenotypic changes observed in polymicrogyria.

### Autism Spectrum Disorder

Also autism spectrum disorders have been associated with pathological apoptosis during brain development (Gabriele et al. 2014). Along those lines, postmortem histological analysis indicates a higher cell density and less clearly structured tissue organization in terms of cell locations (Casanova 2007). Moreover, cortical thickness is increased (Khundrakpam et al. 2017). In order to compare with these observations, we simulated a model where A2-type apoptosis is not functional. As a result, a higher cell density is observed. Moreover, the layers are less clearly separated and more neurons are mislocated (Fig. 7*F*, Supplementary Fig. 3*C*). Furthermore, the cortical thickness is slightly increased (~3 vs. 2.7 mm). Overall, changes to both types of apoptosis, that is, either A1-type (in the first stage) or A2-type (in the second stage), can yield architectures reminiscent of certain neurodevelopmental disorders.

### Subcortical Band Heterotopia

Finally, we conducted simulations of layer formation in subcortical band heterotopia (also known as double cortex syndrome), where neuronal migration is affected (Guerrini and Dobyns 2014). To this end, in our simulations, differentiated neurons were attributed a malfunctioning migratory behavior with a 20% chance. Once differentiated, these pathological cells did not migrate radially but remained in the proliferative zone. This resulted in the abnormal presence of neurons in the proliferative zone (Supplementary Fig. 4), which is in agreement with the observations of a deep cellular layer composed of heterotopic neurons (Manent et al. 2009).

## Discussion

The presence of an abundant variety of layered structures is a common property of cortex and vertebrate brains in general. A mechanistic and in-depth understanding of how cortical layers arise is currently lacking. Computational models are a powerful tool to devise new hypotheses and to generate experimentally testable predictions. In this work, we provide a model for the development of cortical layer architectures and demonstrate that it is applicable to different cortical areas and species. To this end, our results are in accordance with the observation that the number of cortical neurons varies significantly across the cortical surface (Herculano-Houzel et al. 2008; Collins 2011). Moreover, we show that a number of key characteristics of neurodevelopmental disorders can be accounted for. Importantly, our proposed model highlights the role of apoptosis in layer formation and the adaptability of the layer architecture. As such, it provides an explanation for the high proportion of cell death during cortical development (>50%).

Conceptually, our work is different from traditional models of biological development, in that, it follows an agent-based approach. Our model does not rely on any prespecified neural architecture, and it is initialized solely from a homogeneous progenitor cell pool and a single extracellular substance gradient that enables correct cell migration along the radial direction in 3D physical space. Hence, the phenotype is generated in a self-organizingway.

It is well established that apoptosis is abundant during cortical development (Thomaidou et al. 1997; Cavallaro 2015). Already in the early days of neuroscience, the differential roles and mechanisms of apoptosis have been noted (Ernst 1926; Glücksmann 1951). Kuan et al. (2000) discuss the involvement of apoptosis not only in cell number control but also in the proper morphogenesis of the nervous system. Along those lines, Yeo and Gautier review regulatory mechanisms of neural programmed cell death (PCD) during vertebrate neural development (Yeo and Gautier 2004), which is different from neurotrophic death in differentiated neurons.

This model assumes that apoptosis during development comprises differential complexities; in an early apoptotic stage, cell death occurs independently from the local extracellular environment and helps generate roughly the final cell numbers (A1-type). After layer formation has occurred, (A2-type) apoptosis serves a different role by improving the segregation between layers, hence reducing the overlap among layers. Here, the signals from neighboring cells that convey information on the suitability of cell positions within the extracellular context are crucial. In our model, A2-type apoptosis is not necessary for the development of the layer architecture, but it improves the segregation between layers. Notably, two distinct types of apoptosis have been observed experimentally (Rakic and Zecevic 2000), which could correspond to A1-type and A2-type apoptosis. Moreover, it is well established that apoptosis can be induced during development by signaling cascades triggered by neighboring cells (Eroglu and Derry 2016) as is assumed in our proposed model of A2-type apoptosis. It should be possible to experimentally verify the existence of these two hypothetical forms of apoptosis. For instance, A2-type apoptosis could be tested by displacing individual cells during critical developmental stages. Cerebral organoids would offer a suitable testbed for such experiments (Qian et al. 2020).

Importantly, the inclusion of A1-type apoptosis in our model enables a much wider variety of cortical layer architectures. If there was no such apoptosis, changes to the differentiation of early developing layers (e.g., to parameter *C*_6_ in Fig. 1) would necessarily impact all later-developing layers because these early differences influence the number of available progenitor cells and thereby the subsequent differentiation process. Here, we show that apoptosis during the developmental process grants independence to the later-developing layers to evolve differently from the earlier developing ones. Hence, the number of cells in all layers can increase or decrease independently from other layers thicknesses, and so, apoptosis strongly improves this flexibility of the cortical layer architecture. Moreover, our model aligns with the observations of compensatory regulation between apoptosis and proliferation during embryonic cortical development (Freret-Hodara et al. 2017).

We highlight that certain observations naturally follow from our model without tuning of model parameters. Along those lines, experimental work has demonstrated significant rates of apoptosis during proliferation (corresponding to A1-type apoptosis in our model) (Blaschke et al. 1996; Thomaidou et al. 1997). Indeed, also in our simulations, the occurrence of A1-type apoptosis is comparable to the number of generated neurons (Supplementary Fig. 5). Moreover, the rate of embryonic cell death exhibits significant variation across time. This behavior matches with the dynamics of our GRN model (Fig. 1), where apoptotic probabilities of the A1-type apoptosis depend on the GRN state. It is also in accordance with our model that apoptotic rates during development of homologous neuronal populations can vary significantly between species as well as within the same species (Williams and Herrup 1988). Finally, it is established that apoptosis plays a crucial role in determining cortical thickness (Inglis-Broadgate et al. 2005).

Our model suggests that the timing of apoptosis is a crucial factor in the generation of the respective outcome, and so, very different phenotypic properties in neurodevelopmental disorders can be generated depending on the time of apoptotic malfunction. This indicates that certain developmental brain disorders could in fact stem from causes that share many features but occur at different times. Indeed, a number of studies demonstrate the involvement of aberrant apoptosis in developmental brain disorders, such as in autism (Margolis et al. 1994; Wei et al. 2014), FASD (Creeley and Olney 2013), schizophrenia (Glantz et al. 2006), or microcephaly (Poulton et al. 2011). In addition to apoptosis, proliferative dynamics are crucial in generating the appropriate cell numbers and cell types (Lui et al. 2011). Nevertheless, distinct brain developmental disorders will affect proliferation and apoptosis to different extents and can exhibit significant heterogeneity (Donovan and Basson 2017). Hence, the genetic causes can vary depending on the subtype of the developmental disorder, the specific brain region, and the overall genetic context. Notably, specific changes to the proposed GRN model dynamics produce phenotypic outcomes that are in agreement with the observations of healthy and pathological cortical tissue: Changes to apoptosis early during development (A1-type) lead to thinner cortices (polymicrogyria), while introducing a defect to apoptosis later during development (i.e., A2-type apoptosis occurring after migration) gives rise to a more disorganized layer structure associated with autism. Migration deficits yielded pathological gray matter below deep cortical layers, which is in agreement with subcortical band heterotopia.

These findings are relevant from a clinical point of view. One common feature of developmental malformations is a difference in the cortical thickness in various regions. Our model shows the importance of apoptosis in the control of appropriate layer formation and cortical cytoarchitecture. Given this central role of apoptosis, medical intervention to redirect apoptotic behavior at suitable times during brain development is likely a fruitful direction for clinical research. Moreover, for substances that are administered during pregnancy or commonly used in early childhood, particular attention should be attributed to the apoptotic impact (Palanisamy 2012; Sinner et al. 2014). Indeed, reducing apoptosis leads to severe cortical malformation (Kuida et al. 1998). However, increased apoptosis is also a possible origin of disorder (Lainhart and Lange 2011).

Studies of polymicrogyria show increased apoptosis and a thinner cortex (Stottmann et al. 2013; Huang et al. 2016). We find that specific defects in gene regulatory dynamics controlling apoptosis yield characteristic properties associated with this disorder. Intriguingly, the gene *GPR56* has been associated with polymicrogyria (Bahi-Buisson et al. 2010) as well as with apoptotic pathways (Ke et al. 2007; Yang and Xu 2012). Hence, our results are in agreement with the suggestion that polymicrogyria is not a disorder of neuronal migration (Judkins et al. 2011) and highlight the possibility that impaired apoptosis is a key player in the origins of certain neurodevelopmental disorders. However, in our simulations, we introduced a single change to apoptosis occurring before cell migration is induced. Therefore, in contrast to Judkins et al. (2011), we do not suggest that polymicrogyria should be considered a postmigration malformation of cortical development. Instead, we propose that pathological apoptosis before and during migration could be the key driving cause.

Multiple studies demonstrate a tight link between local cytoarchitecture and global brain connectivity as defined by interregional, long-range fiber tracts (Hilgetag and Amunts 2016; Wei et al. 2018). In particular, regions of similar cytoarchitecture and laminar profile tend to be more likely connected. While there are multiple possible explanations for this relationship, it suggests that alterations of the layered cytoarchitecture due to neurodevelopmental disorders will have impact on local as well as long-range connectivity. We anticipate that computational models bridging the local (Bauer et al. 2014) and global (Bauer and Kaiser 2017) spatial scales will help better understand the intricate relationship between cortical layer formation and connectivity.

Given that key neurodevelopmental genes are often involved in many functions (Manzini and Walsh 2011), a specific gene variant might operate correctly at certain developmental stages but may be wrongly expressed at others. Future treatments will need to take into account the specific timing of the genetic expression during development. Along those lines, we envisage that computational models of developmental processes will constitute an additional pillar for clinical research, allowing to anticipate fruitful experiments and formulate novel hypotheses.

Our proposed computational model gives rise to a number of experimentally verifiable and quantifiable predictions. Although the values of the model parameters are underconstrained and do not necessarily match with the true apoptotic rates, the model yields a number of experimentally verifiable predictions. In particular, apoptotic rates should be a major determinant of differences in layer-specific neuron numbers across species as well as within the same species. This is in agreement with previous studies on human and hamster cortex, suggesting that apoptosis could have a key role in shaping regional differences (Finlay and Slattery 1983; Rakic and Zecevic 2000). Along those lines, our model suggests that apoptotic rates change significantly depending on the layer that is being formed. Furthermore, our work suggests that it should be possible, by changing apoptotic events during brain development, to differentially impact cortical layers. In other words, the number of cells in individual layers should be controllable by transient changes to apoptotic rates during proliferation. Such relationship could be assessed experimentally by analyzing markers of apoptosis at different developmental stages during cortical layer formation in combination with layer-specific markers (Hevner 2007; Brunjes and Osterberg 2015). Finally, the computational model suggests that apoptosis, after cell migration has terminated, is relatively large for MZ cells. This is partially due the likelihood of not being pushed by later-developing layers, which is larger than for other layers (see Fig. 6). Additionally, in order to reproduce the small layer 1 neuron numbers, our model requires specifically high MZ cell apoptosis rates in the superficial layers. Indeed, experimental analyses are in agreement with frequent MZ cell apoptosis (Meyer and González-Gómez 2017).

From an evolutionary perspective, our work contributes to a better understanding of a longstanding conundrum on the question why there is so much apoptosis during brain development, that is, approximately one-half of all neurons (Cavallaro 2015). One possibility is that it enables cell selection, that is, inappropriate cells can be removed for the sake of a more refined cortical structure (Pompeiano et al. 2000). However, from an energetic point of view, such massive apoptosis would be surprisingly inefficient. We propose that one key function of apoptosis during brain development is to render the neural architecture more evolvable, enabling the brain to adapt faster and more efficiently to different requirements. Along those lines, it has been previously suggested that apoptosis is responsible for the rapid evolutionary reduction in neuron populations in Spanish wildcats and their domestic relatives (Williams et al. 1993). Moreover, it is in accordance with this evolutionary perspective that apoptosis seems to operate in all multicellular animals (Ameisen 2002). Finally, apoptosis is known to enable greater flexibility as certain evolutionary old structures, such as, for example, pronephric kidney tubules, which form functioning kidneys in fish and in amphibian larvae, are not active in mammals and degenerate (Meier et al. 2000). As mentioned before, it is the appropriate balance between apoptosis and proliferation that is crucial to generate healthy neural structures. Our model suggests that human brain evolution was enabled by a suitable composition of proliferation that is in line with area-specific apoptosis, which determines the properties of the ontogenetic column (Geschwind and Rakic 2013).

Any computational model is based on approximations and so does not reflect many aspects of the actual biological system. We acknowledge that neuronal survival during cortical development is impacted by electrical activity (Heck et al. 2008; Smith and Walsh 2020). Notably, postnatal electrical activity controls neuronal apoptosis in mouse cortex in an area-specific and layer-specific manner (Blanquie et al. 2017). However, it is beyond the scope of this work to cover the full developmental extent of apoptosis in cortex. Cortical layers 1–6 develop before the ingrowth of thalamic afferents, development of corticofugal projections to their targets, and formation of intracortical networks (Huttenlocher and Dabholkar 1997; Kostovic 2002).

It is known that electrical activity is also involved in cortical development during early stages (Spitzer 2006; Yu et al. 2012). Recent work indicates that electrical cellular activation can contribute to migration pausing, eventual arrest, and subsequent dendritogenesis (Medvedeva and Pierani 2020). Moreover, axonal innervation regulates the survival and proliferation of neural progenitor cells in the early developing cortex (Popovik and Haynes 2000). However, there is no evidence that these processes specify area- and layer-specific neuron numbers. We assume that electrical activity during early cortical development, that is, before axons, dendrites, and chemical synapses form, has a permissive rather than instructive role in the formation of area-specific layer architecture. In other words, our working hypothesis is that while electrical activity is necessary for normal proliferation and migration during cortical layer formation, it does not specify the layer architecture. In the future, taking into account additional developmental mechanisms will contribute toward an integrated study of the physiological implications of normal and pathological brain development. Due to their important contribution to the cortical volume, modeling glial cells, neurites, and synapses will also improve accordance with the measurements of cortical neuronal density and layer thickness.

Our model does not include radial glial fibers (RGFs), that is, the processes spanning along the full width of the developing central nervous system. RGFs play important roles during cortical development. They provide a scaffold for the migration of projection neurons that transforms into a physically discontinuous structure during layer formation (Rakic 1972; Nowakowski et al. 2016). In our model, the RGFs’ guidance function is taken over by an initial extracellular gradient that acts as a guidance cue. Moreover, the transient presence of RG subtypes is simplified by the (transient) stem cell types in our proposed core GRN (Fig. 1*B*). As in the case of electrical activity, we assume that RGFs are necessary but not instructive for the specification of the area-specific layer architecture. Along those lines, we did not differentiate between the excitatory and inhibitory neurons in our model. Inhibitory neurons make up between 20% and 30% of the neuronal population, with significant differences across species (Sultan and Shi 2018). However, there is no evidence that differential distribution of inhibitory neurons is the main cause for area or layer differences in neuron numbers. This is further supported by the fact that layer-specific cell numbers can differ by far more than 30% across cortical areas (Charvet et al. 2015). We did not include the development of subplate cells and preplate splitting (Marin-Padilla 1978). The role of the subplate in cortical development is complex and not yet fully understood (Molnár et al. 2019). Hence, its inclusion in the model would render the simulations more time consuming, without adding significant explanatory power relevant to the questions addressed here. A proof-of-principle for the capability to include preplate splitting in our model is shown in Supplementary Figure 6.

We are taking steps toward a comprehensive computational model of cortical development, incorporating neuronal self-organization of biologically plausible axonal arborizations (Bauer et al. 2012; Kassraian-Fard et al. 2020), complex network properties of synaptic connectivity (Bauer and Kaiser 2017), canonical microcircuit function (Bauer et al. 2014), and thalamocortical projections (Kassraian-Fard et al. 2020). We also develop modern coding techniques and software tools (Bauer et al. 2017; Gonzalez de Aledo et al. 2018; Breitwieser et al. 2020), which will enable simulations that give rise to a detailed model of neural tissue dynamics including folding patterns (Wang et al. 2016). Given that our model can account for a wide range of layered neocortical cytoarchitecture, only small amendments are likely to be necessary to explain also other layered structures in the central nervous system, such as the retina (Mellough et al. 2019). In the future, our model could be extended by integrating data on gene expression, such as obtained from public databases (e.g., Polioudakis et al. 2019; Telley et al. 2019). Such detail will enable the modeling of the relationship between genetic specification and the associated phenotypic properties of cortex. Due to the large quantities and complexities, modern machine learning techniques could be employed to produce a tight match with the experimental data. Moreover, we acknowledge the importance of reproducibility and replicability and the benefits of adhering to certain guidelines and approaches (Manninen et al. 2018; Crook et al. 2020). Hence, we provide the associated Cx3Dp code to facilitate the reproduction of our findings.

Overall, we provide a mechanistic, agent-based, and generic computational model for cortical layer formation that takes into account the interaction of intracellular dynamics with extracellular communication. In particular, our model implicates the role of apoptosis as a highly heterogeneous control mechanism, enabling the adaptability and variety of cortical layer structure. Moreover, it constitutes a platform for the generation and testing of hypotheses, such as, for example, on the malfunctioning mechanisms that give rise to developmental disorders.

## Funding

Engineering and Physical Sciences Research Council (EPSRC) of the United Kingdom (EP/K026992/1) as part of the Human Brain Development Project (http://www.greenbrainproject.org/); Medical Research Council (MRC) of the UK (MR/N015037/1 to R.B.); the EPSRC (EP/S001433/1 to R.B.); Wellcome Trust (102037 to M.K.), EPSRC (NS/A000026/1, EP/N031962/1); MRC (MR/T004347/1); and the Guangci Professorship Program of Ruijin Hospital (Shanghai Jiao Tong University).

## Notes

The authors wish to thank Rodney Douglas, Rob Forsyth, Evelyne Sernagor, Peter Taylor, and Yujiang Wang for stimulating discussions and useful comments. This work made use of the facilities of N8 HPC Centre of Excellence, provided and funded by the N8 consortium and EPSRC (grant no. EP/K000225/1). The Centre is co-ordinated by the Universities of Leeds and Manchester. Moreover, parts of the computational work were performed using HPC infrastructure at the Newcastle University and the University of Surrey. *Conflict of Interest:* None declared.

## Data Availability

The simulation code can be found on ModelDB (http://modeldb.yale.edu/266895) and Zenodo (https://zenodo.org/record/4456046#.YAoAMXb7S90).

## Supplementary Material

supplementary_information_bhab003

SupplVideo_S1_bhab003

## References

[ref1] Adesnik H , Naka A. 2018. Cracking the function of layers in the sensory cortex. Neuron. 100:1028–1043. 30521778 10.1016/j.neuron.2018.10.032 PMC6342189

[ref2] Ameisen JC . 2002. On the origin, evolution, and nature of programmed cell death: a timeline of four billion years. Cell Death Differ. 9:367–393. 11965491 10.1038/sj.cdd.4400950

[ref3] Bahi-Buisson N , Poirier K, Boddaert N, Fallet-Bianco C, Specchio N, Bertini E, Caglayan O, Lascelles K, Elie C, Rambaud J, et al. 2010. *GPR56*-related bilateral frontoparietal polymicrogyria: further evidence for an overlap with the cobblestone complex. Brain. 133:3194–3209. 20929962 10.1093/brain/awq259

[ref4] Barkovich AJ , Kuzniecky RI, Jackson GD. 2005. A developmental and genetic classification for malformations of cortical development. Neurology. 65:1873–1887. 16192428 10.1212/01.wnl.0000183747.05269.2d

[ref5] Bauer R , Breitwieser L, Di Meglio A, Johard L, Kaiser M, Manca M, Tchitchigin AD. 2017. The BioDynaMo project: experience report. In: Advanced research on biologically inspired cognitive architectures. Hershey, Pennsylvania, United States: IGI Global, pp. 117–125.

[ref6] Bauer R , Kaiser M. 2017. Nonlinear growth: an origin of hub organization in complex networks. R Soc Open Sci. 4:160691. 28405356 10.1098/rsos.160691 PMC5383813

[ref7] Bauer R , Zubler F, Hauri A, Muir DR, Douglas RJ. 2012. Developmental origin of patchy axonal connectivity in the neocortex: a computational model. Cereb Cortex. 24:487–500. 23131803 10.1093/cercor/bhs327 PMC3888370

[ref8] Bauer R , Zubler F, Pfister S, Hauri A, Pfeiffer M, Muir DR, Douglas RJ. 2014. Developmental self-construction and -configuration of functional neocortical neuronal networks. PLoS Comput Biol. 10:e1003994. 25474693 10.1371/journal.pcbi.1003994 PMC4256067

[ref9] Blanquie O , Yang JW, Kilb W, Sharopov S, Sinning A, Luhmann HJ. 2017. Electrical activity controls area-specific expression of neuronal apoptosis in the mouse developing cerebral cortex. Elife. 2017:e27696. 10.7554/eLife.27696 PMC5582867 28826501

[ref10] Blaschke AJ , Staley K, Chun J. 1996. Widespread programmed cell death in proliferative and postmitotic regions of the fetal cerebral cortex. Development. 122:1165–1174. 8620843 10.1242/dev.122.4.1165

[ref11] Bortone DS , Olsen SR, Scanziani M. 2014. Translaminar inhibitory cells recruited by layer 6 corticothalamic neurons suppress visual cortex. Neuron. 82:474–485. 24656931 10.1016/j.neuron.2014.02.021 PMC4068343

[ref12] Bozzi Y , Casarosa S, Caleo M. 2012. Epilepsy as a neurodevelopmental disorder. Front Psych. 3:1–14. 10.3389/fpsyt.2012.00019 PMC3306997 22457654

[ref13] Breitwieser L , Hesam A, de Montigny J, Vavourakis V, Iosif A, Jennings J, Kaiser M, Manca M, Di Meglio A, Al-Ars Z, et al. 2020. BioDynaMo: an agent-based simulation platform for scalable computational biology research. 1–23.

[ref14a] Brodmann K. 1909. Vergleichende Lokalisationslehre der Grosshirnrinde in ihren Prinzipien dargestellt auf Grund des Zellenbaues. Barth.

[ref14] Brunjes PC , Osterberg SK. 2015. Developmental markers expressed in neocortical layers are differentially exhibited in olfactory cortex. PLoS One. 10:1–23. 10.1371/journal.pone.0138541 PMC4583488 26407299

[ref15] Bystron I , Blakemore C, Rakic P. 2008. Development of the human cerebral cortex: boulder committee revisited. Nat Rev Neurosci. 9(2):110–122. 18209730 10.1038/nrn2252

[ref16] Caffrey JR , Hughes BD, Britto JM, Landman KA. 2014. An in Silico agent-based model demonstrates reelin function in directing lamination of neurons during cortical development. PLoS One. 9:1–11. 10.1371/journal.pone.0110415 PMC4204858 25334023

[ref17] Cahalane DJ , Charvet CJ, Finlay BL. 2014. Modeling local and cross-species neuron number variations in the cerebral cortex as arising from a common mechanism. Proc Natl Acad Sci USA. 111:17642–17647. 25422426 10.1073/pnas.1409271111 PMC4267349

[ref17a] Cajal SR. 1891. Sur la structure de l'écorce cérébrale de quelques mammifères. Cellule. 7:123–176.

[ref18] Casanova MF . 2007. The neuropathology of autism. Mol Basis Autism. 17:153–171. 10.1111/j.1750-3639.2007.00100.x PMC8095561 17919128

[ref19] Cavallaro S . 2015. Cracking the code of neuronal apoptosis and survival. Cell Death Dis. 6:e1963. 26539910 10.1038/cddis.2015.309 PMC4670922

[ref20] Charvet CJ , Cahalane DJ, Finlay BL. 2015. Systematic, cross-cortex variation in neuron numbers in rodents and primates. Cereb Cortex. 25(1):147–160. 23960207 10.1093/cercor/bht214 PMC4259279

[ref21] Collins CE . 2011. Variability in neuron densities across the cortical sheet in primates. Brain Behav Evol. 78:37–50. 21691046 10.1159/000327319

[ref22] Crawley JN . 2012. Translational animal models of autism and neurodevelopmental disorders. Dialogues Clin Neurosci. 14:293–305. 23226954 10.31887/DCNS.2012.14.3/jcrawley PMC3513683

[ref23] Creeley CE , Olney JW. 2013. Drug-induced apoptosis: mechanism by which alcohol and many other drugs can disrupt brain development. Brain Sci. 3:1153–1181. 24587895 10.3390/brainsci3031153 PMC3938204

[ref24] Crook SM , Davison AP, McDougal RA, Plesser HE. 2020. Editorial: reproducibility and rigour in computational neuroscience. Front Neuroinform. 14:23. 10.3389/fninf.2020.00023 PMC7267030 32536859

[ref25] Cuntz H , Forstner F, Borst A, Häusser M. 2010. One rule to grow them all: a general theory of neuronal branching and its practical application. PLoS Comput Biol. 6:e1000877. 20700495 10.1371/journal.pcbi.1000877 PMC2916857

[ref26] DeFelipe J , Alonso-Nanclares L, Arellano JI. 2002. Microstructure of the neocortex: comparative aspects. J Neurocytol. 31:299–316. 12815249 10.1023/a:1024130211265

[ref27] Donovan APA , Basson MA. 2017. The neuroanatomy of autism—a developmental perspective. J Anat. 230:4–15. 27620360 10.1111/joa.12542 PMC5192959

[ref28] Douglas RJ , Martin KAC. 2004. Neural circuits of the neocotex. Annu Rev Neurosci. 27:419–451. 15217339 10.1146/annurev.neuro.27.070203.144152

[ref29] Douglas RJ , Martin KAC, Whitteridge D. 1989. A canonical microcircuit for Neocortex. Neural Comput. 1:480–488.

[ref31] Eiraku M , Sasai Y. 2012. Self-formation of layered neural structures in three-dimensional culture of ES cells. Curr Opin Neurobiol. 22:768–777. 22405989 10.1016/j.conb.2012.02.005

[ref32] Ernst M . 1926. Über Untergang von Zellen während der normalen Entwicklung bei Wirbeltieren. Z Anat Entwickl Gesch. 79:228–262.

[ref33] Eroglu M , Derry WB. 2016. Your neighbours matter-non-autonomous control of apoptosis in development and disease. Cell Death Differ. 23(7):1110–1118. 10.1038/cdd.2016.41 PMC4946894 27177021

[ref34] Finlay BL , Slattery M. 1983. Local differences in the amount of early cell death in neocortex predict adult local specializations. Science (80-). 219:1349–1351. 10.1126/science.6828866 6828866

[ref35] Freret-Hodara B , Cui Y, Griveau A, Vigier L, Arai Y, Touboul J, Pierani A. 2017. Enhanced Abventricular proliferation compensates cell death in the embryonic cerebral cortex. Cereb Cortex. 27(10):4701–4718. 10.1093/cercor/bhw264 27620979

[ref36] Gabriele S , Lombardi F, Sacco R, Napolioni V, Altieri L, Tirindelli MC, Gregorj C, Bravaccio C, Rousseau F, Persico AM. 2014. The GLO1 C332 (Ala111) allele confers autism vulnerability: family-based genetic association and functional correlates. J Psychiatr Res. 59:108–116. 25201284 10.1016/j.jpsychires.2014.07.021

[ref37] Gao P , Postiglione MP, Krieger TG, Hernandez L, Wang C, Han Z, Streicher C, Papusheva E, Insolera R, Chugh K, et al. 2014. Deterministic progenitor behavior and unitary production of neurons in the neocortex. Cell. 159:775–788. 25417155 10.1016/j.cell.2014.10.027 PMC4225456

[ref38] García-Cabezas MÁ , Zikopoulos B. 2019. Evolution, development, and organization of the cortical connectome. PLoS Biol. 17(5): e3000259 10.1371/journal.pbio.3000259 PMC6530863 31075099

[ref39] Gaspard N . 2011. Laminar fate specification in the cerebral cortex. F1000 Biol Rep. 3:1–6. 21655334 10.3410/B3-6 PMC3100784

[ref40] Geschwind DH , Rakic P. 2013. Cortical evolution: judge the brain by its cover. Neuron. 80:633–647. 24183016 10.1016/j.neuron.2013.10.045 PMC3922239

[ref41] Glantz LA , Gilmore JH, Lieberman JA, Jarskog LF. 2006. Apoptotic mechanisms and the synaptic pathology of schizophrenia. Schizophr Res. 81:47–63. 16226876 10.1016/j.schres.2005.08.014

[ref42] Glücksmann A . 1951. Cell deaths in normal vertebrate ontogeny. Biol Rev. 26:59–86. 24540363 10.1111/j.1469-185x.1951.tb00774.x

[ref43] Golden JA , Harding BN. 2010. Cortical malformations: unfolding polymicrogyria. Nat Rev Neurol. 6:471. 20811463 10.1038/nrneurol.2010.118

[ref44] Gonzalez de Aledo P , Vladimirov A, Manca M, Baugh J, Asai R, Kaiser M, Bauer R. 2018. An optimization approach for agent-based computational models of biological development. Adv Eng Softw. 121:262–275.

[ref45] Goulas A , Majka P, Rosa MGP, Hilgetag CC. 2019. A blueprint of mammalian cortical connectomes. PLoS Biol. 17(3):e2005346. 10.1371/journal.pbio.2005346 PMC6456226 30901324

[ref46] Guerin MB , McKernan DP, O’Brien CJ, Cotter TG. 2002. Retinal ganglion cells: dying to survive. Int J Dev Biol. 50:665–674. 10.1387/ijdb.062159mg 17051476

[ref47] Guerrini R , Dobyns WB. 2014. Malformations of cortical development: clinical features and genetic causes. Lancet Neurol. 13:710–726. 24932993 10.1016/S1474-4422(14)70040-7 PMC5548104

[ref48] Hauri A . 2013. Self-construction in the context of cortical growth: from one cell to a cortex to a programming paradigm for self-constructing systems [PhD thesis]. Switzerland: ETH Zurich.

[ref49] Heck N , Golbs A, Riedemann T, Sun J-J, Lessmann V, Luhmann HJ. 2008. Activity-dependent regulation of neuronal apoptosis in neonatal mouse cerebral cortex. Cereb Cortex. 18:1335–1349. 17965127 10.1093/cercor/bhm165

[ref50] Herculano-Houzel S , Collins CE, Wong P, Kaas JH, Lent R. 2008. The basic nonuniformity of the cerebral cortex. Proc Natl Acad Sci USA. 105:12593–12598. 18689685 10.1073/pnas.0805417105 PMC2527956

[ref51] Hevner RF . 2007. Layer-specific markers as probes for neuron type identity in human neocortex and malformations of cortical development. J Neuropathol Exp Neurol. 66:101–109. 17278994 10.1097/nen.0b013e3180301c06

[ref52] Hilgetag CC , Amunts K. 2016. Connectivity and cortical architecture (Konnektivität und kortikale Architektur). e-Neuroforum. 7:56–63.

[ref53] His W . 1904. Die Entwickelung des menschlichen Gehirns: waehrend der ersten Monate. Stuttgart, Germany: S. Hirzel.

[ref54] Huang WC , Abraham R, Shim BS, Choe H, Page DT. 2016. Zika virus infection during the period of maximal brain growth causes microcephaly and corticospinal neuron apoptosis in wild type mice. Sci Rep. 6:1–8. 27713505 10.1038/srep34793 PMC5054421

[ref55] Huttenlocher PR , Dabholkar AS. 1997. Regional differences in synaptogenesis in human cerebral cortex. J Comp Neurol. 387:167–178. 9336221 10.1002/(sici)1096-9861(19971020)387:2<167::aid-cne1>3.0.co;2-z

[ref56] Inglis-Broadgate SL , Thomson RE, Pellicano F, Tartaglia MA, Pontikis CC, Cooper JD, Iwata T. 2005. FGFR3 regulates brain size by controlling progenitor cell proliferation and apoptosis during embryonic development. Dev Biol. 279:73–85. 15708559 10.1016/j.ydbio.2004.11.035

[ref57] Judkins AR , Martinez D, Ferreira P, Dobyns WB, Golden JA. 2011. Polymicrogyria includes fusion of the molecular layer and decreased neuronal populations but normal cortical laminar organization. J Neuropathol Exp Neurol. 70:438–443. 21572338 10.1097/NEN.0b013e31821ccf1c PMC3113653

[ref58] Kassraian-Fard P , Pfeiffer M, Bauer R. 2020. A generative growth model for thalamocortical axonal branching in primary visual cortex. PLoS Comput Biol. 16(2):e1007315. 10.1371/journal.pcbi.1007315 PMC7018004 32053598

[ref59] Ke N , Sundaram R, Liu G, Chionis J, Fan W, Rogers C, Awad T, Grifman M, Yu D, Wong-Staal F, et al. 2007. Orphan G protein-coupled receptor *GPR56* plays a role in cell transformation and tumorigenesis involving the cell adhesion pathway. Mol Cancer Ther. 6:1840–1850. 17575113 10.1158/1535-7163.MCT-07-0066

[ref60] Khundrakpam BS , Lewis JD, Kostopoulos P, Carbonell F, Evans AC. 2017. Cortical thickness abnormalities in autism spectrum disorders through late childhood, adolescence, and adulthood: a large-scale MRI study. Cereb Cortex. 27(3):1721–1731. 10.1093/cercor/bhx038 28334080

[ref61] Koene RA , Tijms B, Van Hees P, Postma F, De Ridder A, Ramakers GJA, Van Pelt J, Van Ooyen A. 2009. NETMORPH: a framework for the stochastic generation of large scale neuronal networks with realistic neuron morphologies. Neuroinformatics. 7:195–210. 19672726 10.1007/s12021-009-9052-3

[ref62] Kostovic I . 2002. Laminar organization of the human fetal cerebrum revealed by histochemical markers and magnetic resonance imaging. Cereb Cortex. 12:536–544. 11950771 10.1093/cercor/12.5.536

[ref62a] Kuan CY, Roth KA, Flavell RA, Rakic P. 2000. Mechanisms of programmed cell death in the developing brain. Trends Neurosci. 23(7):291–297. 10856938 10.1016/s0166-2236(00)01581-2

[ref63] Kuhlenbeck H . 1967. The central nervous system of vertebrates: a general survey of its comparative anatomy with an introd. to the pertinent fundamental biologic and logical concepts. Basel, Switzerland: Karger Medical and Scientific Publishers.

[ref64] Kuida K , Haydar TF, Kuan CY, Gu Y, Taya C, Karasuyama H, Su MSS, Rakic P, Flavell RA. 1998. Reduced apoptosis and cytochrome C-mediated caspase activation in mice lacking Caspase 9. Cell. 94:325–337. 9708735 10.1016/s0092-8674(00)81476-2

[ref65] Lainhart JE , Lange N. 2011. Increased neuron number and head size in autism. JAMA. 306:2031–2032. 22068999 10.1001/jama.2011.1633

[ref66] Lancaster MA , Knoblich JA. 2014. Organogenesis in a dish: modeling development and disease using organoid technologies. Science (80-). 345:1247125. 10.1126/science.1247125 25035496

[ref67] Lancaster MA , Renner M, Martin CA, Wenzel D, Bicknell LS, Hurles ME, Homfray T, Penninger JM, Jackson AP, Knoblich JA. 2013. Cerebral organoids model human brain development and microcephaly. Nature. 501:373–379. 23995685 10.1038/nature12517 PMC3817409

[ref68] Larkum ME , Petro LS, Sachdev RNS, Muckli L. 2018. A perspective on cortical layering and layer-spanning neuronal elements. Front Neuroanat. 12:56. 30065634 10.3389/fnana.2018.00056 PMC6056619

[ref68a] Lewis B. 1878. On the comparative structure of the cortex cerebri. Brain. 1(1):79–96.

[ref69] Lui JH , Hansen DV, Kriegstein AR. 2011. Development and evolution of the human neocortex. Cell. 146:18–36. 21729779 10.1016/j.cell.2011.06.030 PMC3610574

[ref70] Lur G , Vinck MA, Tang L, Cardin JA, Higley MJ. 2016. Projection-specific visual feature encoding by layer 5 cortical subnetworks. Cell Rep. 14:2538–2545. 26972011 10.1016/j.celrep.2016.02.050 PMC4805451

[ref71] Macneil LT , Walhout AJM. 2011. Gene regulatory networks and the role of robustness and stochasticity in the control of gene expression. Genome Res. 21:645–657. 21324878 10.1101/gr.097378.109 PMC3083081

[ref72] Manent J-B , Wang Y, Chang Y, Paramasivam M, LoTurco JJ. 2009. Dcx reexpression reduces subcortical band heterotopia and seizure threshold in an animal model of neuronal migration disorder. Nat Med. 15:84. 19098909 10.1038/nm.1897 PMC2715867

[ref73] Manninen T , Aćimović J, Havela R, Teppola H, Linne ML. 2018. Challenges in reproducibility, replicability, and comparability of computational models and tools for neuronal and glial networks, cells, and subcellular structures. Front Neuroinform. 12:20. 10.3389/fninf.2018.00020 PMC5938413 29765315

[ref74] Manzini MC , Walsh CA. 2011. What disorders of cortical development tell us about the cortex: one plus one does not always make two. Curr Opin Genet Dev. 21:333–339. 21288712 10.1016/j.gde.2011.01.006 PMC3139684

[ref75] Margolis RL , Chuang D-M, Post RM. 1994. Programmed cell death: implications for neuropsychiatric disorders. Biol Psychiatry. 35:946–956. 8080894 10.1016/0006-3223(94)91241-6

[ref76] Marin-Padilla M . 1978. Dual origin of the mammalian neocortex and evolution of the cortical plate. Anat Embryol. 152(2):109–126. 10.1007/BF00315920 637312

[ref77] Markov NT , Vezoli J, Chameau P, Falchier A, Quilodran R, Huissoud C, Lamy C, Misery P, Giroud P, Ullman S, et al. 2014. Anatomy of hierarchy: feedforward and feedback pathways in macaque visual cortex. J Comp Neurol. 522:225–259. 23983048 10.1002/cne.23458 PMC4255240

[ref78] Medvedeva VP , Pierani A. 2020. How do electric fields coordinate neuronal migration and maturation in the developing cortex? Front Cell Dev Biol. 8:1006. 10.3389/fcell.2020.580657 PMC7546860 33102486

[ref79] Meier P , Finch A, Evan G. 2000. Apoptosis in development. Nature. 407:796. 11048731 10.1038/35037734

[ref80] Mellough CB , Bauer R, Collin J, Dorgau B, Zerti D, Dolan DWP, Jones CM, Izuogu OG, Yu M, Hallam D, et al. 2019. An integrated transcriptional analysis of the developing human retina. Development. 146(2):dev169474. 10.1242/dev.169474 PMC6361134 30696714

[ref81] Meyer G , González-Gómez M. 2017. The subpial granular layer and transient versus persisting Cajal-Retzius neurons of the fetal human cortex. Cereb Cortex. 28:1–16. 10.1093/cercor/bhx110 28472243

[ref82] Molnár Z , Clowry GJ, Šestan N, Alzu’bi A, Bakken T, Hevner RF, Hüppi PS, Kostović I, Rakic P, Anton ES, et al. 2019. New insights into the development of the human cerebral cortex. J Anat. 235(3):432–451. 10.1111/joa.13055 PMC6704245 31373394

[ref83] Molyneaux BJ , Arlotta P, Menezes JRL, Macklis JD. 2007. Neuronal subtype specification in the cerebral cortex. Nat Rev Neurosci. 8:427–437. 17514196 10.1038/nrn2151

[ref84] Nawa H , Takahashi M, Patterson PH. 2000. Cytokine and growth factor involvement in schizophrenia—support for the developmental model. Mol Psychiatry. 5:594–603. 11126390 10.1038/sj.mp.4000730

[ref85] Nowakowski TJ , Pollen AA, Sandoval-Espinosa C, Kriegstein AR. 2016. Transformation of the radial glia scaffold demarcates two stages of human cerebral cortex development. Neuron. 91(6):1219–1227. 10.1016/j.neuron.2016.09.005 PMC5087333 27657449

[ref86] O’Kusky J , Colonnier M. 1982. A laminar analysis of the number of neurons, glia, and synapses in the adult cortex (area 17) of adult macaque monkeys. J Comp Neurol. 210:278–290. 7142443 10.1002/cne.902100307

[ref87] Palanisamy A . 2012. Maternal anesthesia and fetal neurodevelopment. Int J Obstet Anesth. 21:152–162. 22405978 10.1016/j.ijoa.2012.01.005

[ref88] Polioudakis D , de la Torre-Ubieta L, Langerman J, Elkins AG, Shi X, Stein JL, Vuong CK, Nichterwitz S, Gevorgian M, Opland CK, et al. 2019. A single-cell Transcriptomic atlas of human neocortical development during mid-gestation. Neuron. 103(5):785–801. 10.1016/j.neuron.2019.06.011 PMC6831089 31303374

[ref89] Pompeiano M , Blaschke AJ, Flavell RA, Srinivasan A, Chun J. 2000. Decreased apoptosis in proliferative and postmitotic regions of the caspase 3-deficient embryonic central nervous system. J Comp Neurol. 423:1–12. 10861532

[ref90] Popovik E , Haynes LW. 2000. Survival and mitogenesis of neuroepithelial cells are influenced by noradrenergic but not cholinergic innervation in cultured embryonic rat neopallium. Brain Res. 853:227–235. 10640620 10.1016/s0006-8993(99)02242-8

[ref91] Poulton CJ , Schot R, Kia SK, Jones M, Verheijen FW, Venselaar H, De Wit MCY, De Graaff E, Bertoli-Avella AM, Mancini GMS. 2011. Microcephaly with simplified gyration, epilepsy, and infantile diabetes linked to inappropriate apoptosis of neural progenitors. Am J Hum Genet. 89:265–276. 21835305 10.1016/j.ajhg.2011.07.006 PMC3155199

[ref92] Qian X , Su Y, Adam CD, Deutschmann AU, Pather SR, Goldberg EM, Su K, Li S, Lu L, Jacob F, et al. 2020. Sliced human cortical Organoids for Modeling distinct cortical layer formation. Cell Stem Cell. 26(5):766–781. 10.1016/j.stem.2020.02.002 PMC7366517 32142682

[ref93] Quiquempoix M , Fayad SL, Boutourlinsky K, Leresche N, Lambert RC, Bessaih T. 2018. Layer 2/3 pyramidal neurons control the gain of cortical output. Cell Rep. 24:2799–2807. 30208307 10.1016/j.celrep.2018.08.038

[ref94] Rakic P . 1972. Mode of cell migration to the superficial layers of fetal monkey neocortex. J Comp Neurol. 145(1):61–83. 10.1002/cne.901450105 4624784

[ref94a] Rakic P, Ayoub AE, Breunig JJ, Dominguez MH. 2009. Decision by division: making cortical maps. Trends Neurosci. 32(5):291–301. 10.1016/j.tins.2009.01.007 PMC3601545 19380167

[ref95] Rakic S , Zecevic N. 2000. Programmed cell death in the developing human telencephalon. Eur J Neurosci. 12:2721–2734. 10971615 10.1046/j.1460-9568.2000.00153.x

[ref96] Riley EP , Infante MA, Warren KR. 2011. Fetal alcohol spectrum disorders: an overview. Neuropsychol Rev. 21:73–80. 21499711 10.1007/s11065-011-9166-x PMC3779274

[ref97] Saito T , Hanai S, Takashima S, Nakagawa E, Okazaki S, Inoue T, Miyata R, Hoshino K, Akashi T, Sasaki M, et al. 2011. Neocortical layer formation of human developing brains and lissencephalies: consideration of layer-specific marker expression. Cereb Cortex. 21:588–596. 20624841 10.1093/cercor/bhq125

[ref98] Schüz A , Palm G. 1989. Density of neurons and synapses in the cerebral cortex of the mouse. J Comp Neurol. 286:442–455. 2778101 10.1002/cne.902860404

[ref99] Sinner B , Becke K, Engelhard K. 2014. General anaesthetics and the developing brain: an overview. Anaesthesia. 69:1009–1022. 24829066 10.1111/anae.12637

[ref100] Smith RS , Walsh CA. 2020. Ion Channel functions in early brain development. Trends Neurosci. 43(2):103–114. 10.1016/j.tins.2019.12.004 PMC7092371 31959360

[ref101] Spitzer NC . 2006. Electrical activity in early neuronal development. Nature. 444(7120):707–712. 10.1038/nature05300 17151658

[ref102] Stottmann RW , Donlin M, Hafner A, Bernard A, Sinclair DA, Beier DR. 2013. Amutation in tubb2b, a human polymicrogyria gene, leads to lethality and abnormal cortical development in the mouse. Hum Mol Genet. 22:4053–4063. 23727838 10.1093/hmg/ddt255 PMC3781635

[ref103] Striedter GF . 2005. Principles of brain evolution. Sunderland, Massachusetts, United States: Sinauer Associates.

[ref104] Sultan KT , Shi SH. 2018. Generation of diverse cortical inhibitory interneurons. Wiley Interdiscip Rev Dev Biol. 7(2):e306. 10.1002/wdev.306 PMC5814332 29115042

[ref105] Telley L , Agirman G, Prados J, Amberg N, Fièvre S, Oberst P, Bartolini G, Vitali I, Cadilhac C, Hippenmeyer S, et al. 2019. Temporal patterning of apical progenitors and their daughter neurons in the developing neocortex. Science. 364(6440):eaav2522. 10.1126/science.aav2522 31073041

[ref106] Thomaidou D , Mione MC, Cavanagh JF, Parnavelas JG. 1997. Apoptosis and its relation to the cell cycle in the developing cerebral cortex. J Neurosci. 17:1075–1085. 8994062 10.1523/JNEUROSCI.17-03-01075.1997 PMC6573180

[ref30] von Economo C . 2009. Cellular structure of the human Cerebral Cortex. Basel, Switzerland: Karger Medical and Scientific Publishers.

[ref107] Wang Y , Necus J, Kaiser M, Mota B. 2016. Universality in human cortical folding in health and disease. Proc Natl Acad Sci USA. 113:12820–12825. 27791126 10.1073/pnas.1610175113 PMC5111660

[ref108] Wei H , Alberts I, Li X. 2014. The apoptotic perspective of autism. Int J Dev Neurosci. 36:13–18. 24798024 10.1016/j.ijdevneu.2014.04.004

[ref109] Wei Y , Scholtens LH, Turk E, van den Heuvel MP. 2018. Multiscale examination of cytoarchitectonic similarity and human brain connectivity. Netw Neurosci. 3:124–137. 30793077 10.1162/netn_a_00057 PMC6372019

[ref110] Williams RW , Cavada C, Reinoso-Suárez F. 1993. Rapid evolution of the visual system: a cellular assay of the retina and dorsal lateral geniculate nucleus of the Spanish wildcat and the domestic cat. J Neurosci. 13:208–228. 8423469 10.1523/JNEUROSCI.13-01-00208.1993 PMC6576313

[ref111] Williams RW , Herrup K. 1988. The control of neuron number. Annu Rev Neurosci. 11:423–453. 3284447 10.1146/annurev.ne.11.030188.002231

[ref112] Workman AD , Charvet CJ, Clancy B, Darlington RB, Finlay BL. 2013. Modeling transformations of neurodevelopmental sequences across mammalian species. J Neurosci. 33:7368–7383. 23616543 10.1523/JNEUROSCI.5746-12.2013 PMC3928428

[ref114] Yang L , Xu L. 2012. *GPR56* in cancer progression: current status and future perspective. Future Oncol. 8:431–440. 22515446 10.2217/fon.12.27

[ref115] Yeo W , Gautier J. 2004. Early neural cell death: dying to become neurons. Dev Biol. 274:233–244. 15385155 10.1016/j.ydbio.2004.07.026

[ref116] Yu YC , He S, Chen S, Fu Y, Brown KN, Yao XH, Ma J, Gao KP, Sosinsky GE, Huang K, et al. 2012. Preferential electrical coupling regulates neocortical lineage-dependent microcircuit assembly. Nature. 486(7401):113–117. 10.1038/nature10958 PMC3599787 22678291

[ref117] Zubler F . 2009. A framework for modeling the growth and development of neurons and networks. Front Comput Neurosci. 3:1–16. 19949465 10.3389/neuro.10.025.2009 PMC2784082

[ref118] Zubler F , Hauri A, Pfister S, Bauer R, Anderson JC, Whatley AM, Douglas RJ. 2013. Simulating cortical development as a self constructing process: a novel multi-scale approach combining molecular and physical aspects. PLoS Comput Biol. 9:e1003173. 23966845 10.1371/journal.pcbi.1003173 PMC3744399

[ref119] Zubler F , Hauri A, Pfister S, Whatley AM, Cook M, Douglas R. 2011. An instruction language for self-construction in the context of neural networks. Front Comput Neurosci. 5:57. 10.3389/fncom.2011.00057 PMC3233694 22163218

