# Supplementary material for: Creative Destruction: A Basic Computational Model of Cortical Layer Formation
Source: Cereb Cortex. 2021 Feb 24;31(7):3237–53. doi: 10.1093/cercor/bhab003 (PMC8196252; doi:10.1093/cercor/bhab003)
Supplement: supplementary_information_bhab003 [file supplementary_information_bhab003.pdf]

Supplementary information for "Creative destruction: a basic computational model of cortical layer formation"

1 Software framework

In this study, we employed the software framework *Cx3Dp*. *Cx3Dp* is the parallelised version of *Cx3D* (Zubler and Douglas, Front Comput Neurosci, 2009). Both *Cx3D* and *Cx3Dp* are available as open-source code from `www.ini.uzh.ch/~amw/seco/cx3d/` and are distributed under the GNU General Public License version 3. In the following, the features of the *Cx3D* framework are summarised.

Cx3D is a Java-based tool for simulating the growth of cortex in 3D. It allows to model multiple stages of corticogenesis, including processes such as cell division and migration, extension of axonal and dendritic arbors, and establishment of synaptic connections. Notably, it also allows to take into account 3D physical spye by a physics engine with computes the mechanical forces between objects that are either spheres (e.g. for cell bodies) or cylinders (e.g. for neurite segments and physical bonds).

Each discrete physical component is located at a particular point in 3D space. Each evaluation for a possible interaction between any two objects i has a computational cost. To limit this cost, each object maintains a list of neighbouring objects with which it might interact. This list is updated when an object moves, or when an object is added or deleted from the space. To define the neighborhood relation a 3D Delaunay triangulation is used. Along those lines, substance concentrations and diffusion are represented based on a dual graph, similar to a finite volume method approach.

Somata are defined by a sphere with a central point mass. Their movements are determined by different types of forces. One type arises from the interaction between somata when they come into close contact. Another type represents the active movement of these somata, which follows from the biological behaviours of the model specified by the user. In particular, cells are modelled as a granular medium with additional bindings. The physical interaction between two spherical somata is a function of their diameter, their relative distance and possibly thir xpression of adherence molecules. In addition, there is also a term for static friction that provides a threshold for the initiation of movement. The actual movement is only computed If the total force exceeds a static friction threshold.

2 Layer formation

The video Suppl. Video S1 shows a simulation of layer formation in accordance with spatial dimensions of human temporal cortex with (Fig. 3). An initial, homogeneous precursor pool of proliferating cells gives rise to different neuron types in a given order. Each cell comprises an individual gene-regulatory

network, and interacts with its local external environment. The final cortical column comprises approximately 7,000 neurons. The video can also be found on Youtube: `https://youtu.be/pYxnK3YXQmU`.

3 Layer architecture: parameter variation

Suppl. Fig. S1 shows the impact of changing examplary GRN parameters on the layer architecture.

4 Analysis of inter-layer thickness correlations

We investigated whether there were negative correlations among layer thicknesses, as would be predicted by our model without A1-type apoptosis (assuming that layer thicknesses depend linearly on the number of neurons). Negative correlations would exist in the scenario without A1-type apoptosis because increased layer thickness at one stage would entail a reduced number of later-developing neurons, hence thinner later-developing layers. Suppl. Fig. S2 shows that such negative correlations were not found.

5 Polymicrogyria and Autism Spectrum Disorder

We conducted simulations of a pathological neurodevelopmental scenario. Suppl. Fig. S3 shows how malfunctioning apoptosis during layer formation can give rise to cortical structure reminiscent of certain neurodevelopmental disorders. In Suppl. Fig. S3B, overexpressed apoptosis produes a cortex that is thinner than in the control case. Such a thinning was generated by enhanced apoptosis during the proliferation stage (A1-type apoptosis in Fig. 1). Alternatively, the initial, exponential proliferation phase can be shorted by increasing the threshold for initiation neural differentiation. In this case, the early proliferative phase consisting of symmetric division is shortened, hence fewer neurons are generated. Again, this leads to a thinner cortex.

In a second scenario, A2-type apoptosis was removed from the simulation (see also Fig. 1). This still produced a layered cortex but comprised significant spatial mixing of different cell types, as shown by the overlap among bars of diffrent colors (Suppl. Fig. S3C).

6 Subcortical heterotopia

We also simulated the developmental disorder of subcortical band heterotopia, where heterotopic neurons in the proliferative zone can be observed (Suppl. Fig. S4). Introducing defects to neuronal migration gives rise to characteristics of malformation in agreement with this disorder.

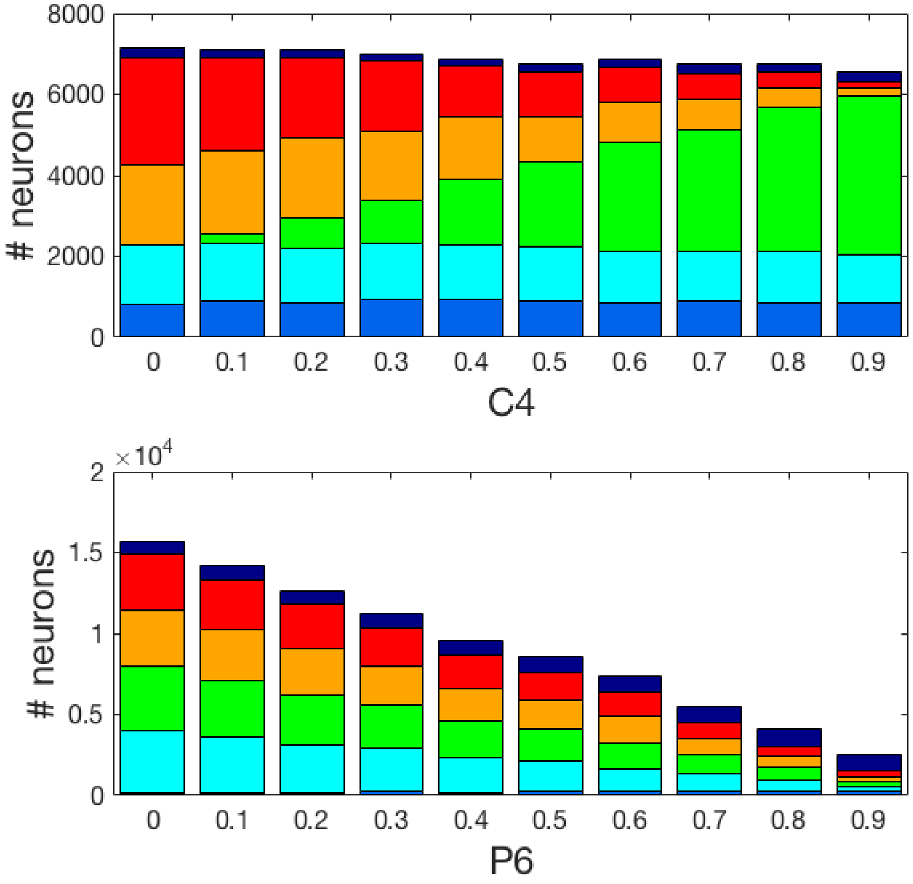

Figure S1: Layer architectures for different gene regulatory network parameter settings. In the upper plot, the probability to commit to layer 4 is varied, showing its impact on the number of neurons of layers 4, 3 and 2. In the bottom plot, the apoptotic probability P6 is varied, influencing the number of neurons across all layers, due to the early activation of this apoptotic process.

7 Occurrence of apoptosis

Our model incorporates two modes of apoptosis, i.e. type A1 and type A2. Type A1 is independent on environmentanl signals, while type A2 depends on the cell types in the local neighborhood of cells. Suppl. Fig. S5 shows example numbers of occurrences of apoptosis in a simulation of layer formation in human temporal cortex. In agreement with experimental data, apoptosis is highly abundant during cortical development.

8 Splitting of preplate into subplate and marginal zone

In addition to the simplified scenario described in the manuscript, where no subplate cells are simulated, we here demonstrate that our model can be extended to take into account the splitting of the preplate into subplate and marginal zone. For the sake of faster simulation, we did not implement this extended model in the simulations described in the main manuscript. Suppl. Fig. S6 shows as a sequence the temporal changes in this updated scenario, where the initial extracllular gradient along the radial direction acts as a guidance cue.

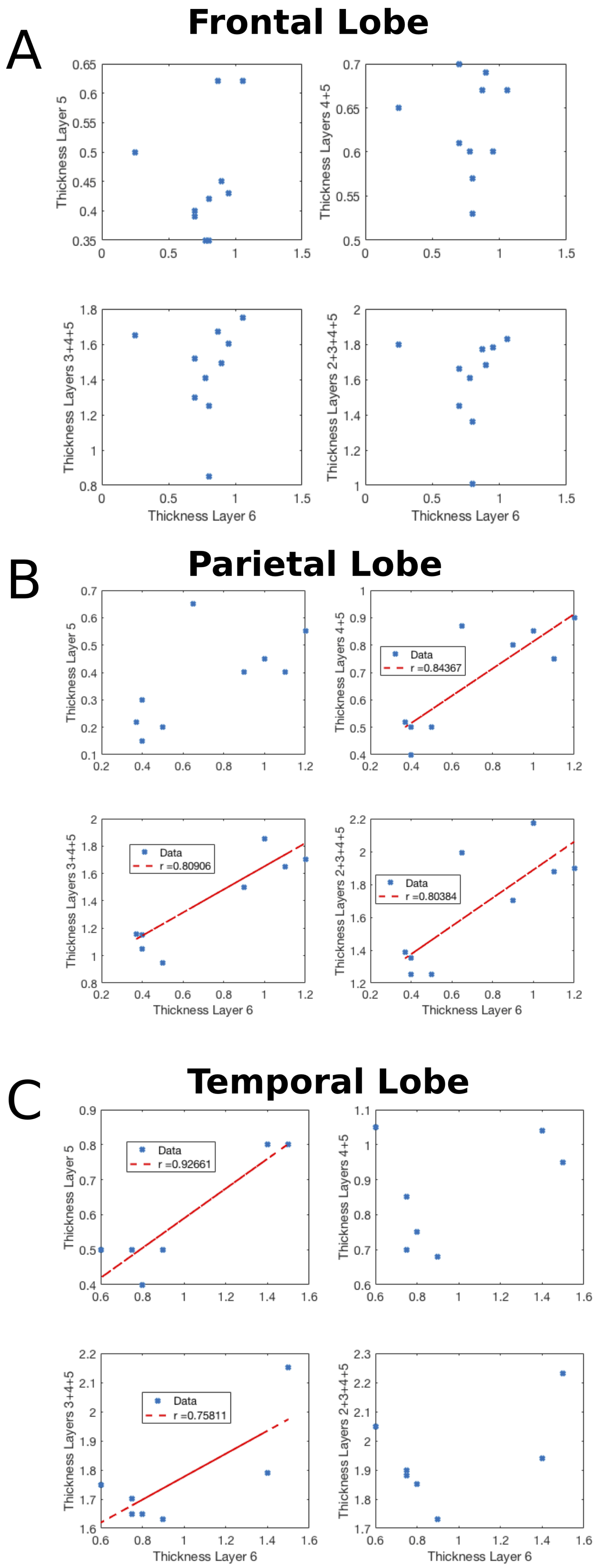

Figure S2: Examination of correlations between layer thicknesses in human cortex, based on data from Economo (2009). Layer thicknesses in areas in the (A) human frontal lobe (B) human parietal lobe (C) human temporal lobe. Red lines indicate statistically significant ($p<0.05$) correlations. There is no evidence that increases or decreases of early developing layers lead to a reduction or promotion, respectively, of late-developing layers.

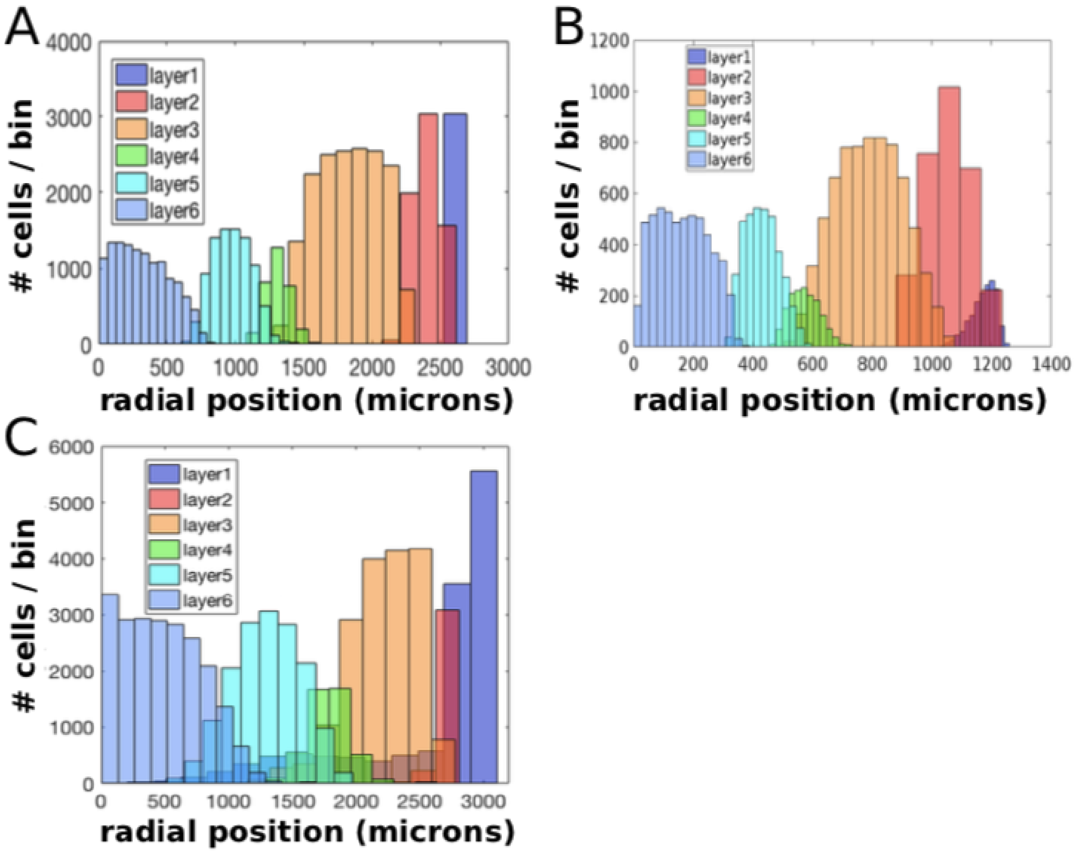

Figure S3: Malfunctioning proliferation or apoptosis during layer formation yields structural features characteristic for neurodevelopmental diseases. (A) As a control, the development of the layer structure in agreement with layer thickness measurements from human peristriate area 19 was simulated. The simulated healthy layer architecture yields cell distributions for the different layers that are well segregated and there is little overlap between cells of neighbouring layers. (B) Certain neurodevelopmental diseases, such as polymigrogyria, are also characterised by a thinner cortex. In our model, by reducing the number of symmetric cell divisions, the resulting layer structure becomes significantly thinner (1.3mm *vs.* 2.7 mm). Also by increasing the amount of A1-type apoptosis, cortical thickness is reduced (Fig. 7E). (C) In our model, when we remove A2-type apoptosis, cortical layers still form. However, there is significant overlap between these, as visualised by the strongly overlapping distribution bar plots. In other words, distinct cell types are spatially mixed within the same layers. Also in autism spectrum disorder, increased mixing of different cell types has been observed.

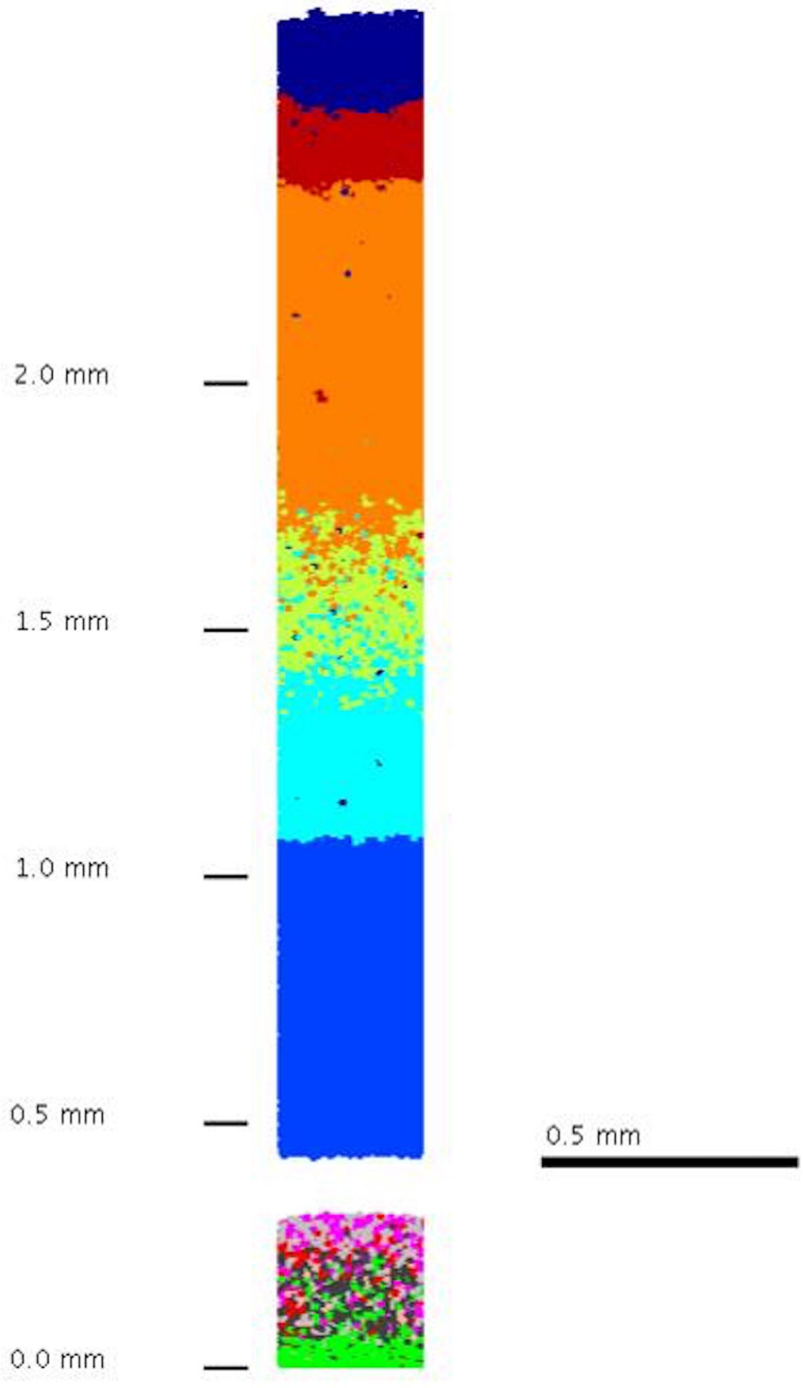

Figure S4: Simulation of cortical organization in subcortical heterotopia. Characteristic features of subcortical heterotopia or double-cortex syndrome are recapitulated by introducing migration defects. Here, 30 % of differentiated neurons exhibited pathological migration and remained in the proliferative zone. As a consequence, heterotopic neurons are observed below the deep cortical layers, while cortical layer structure is preserved.

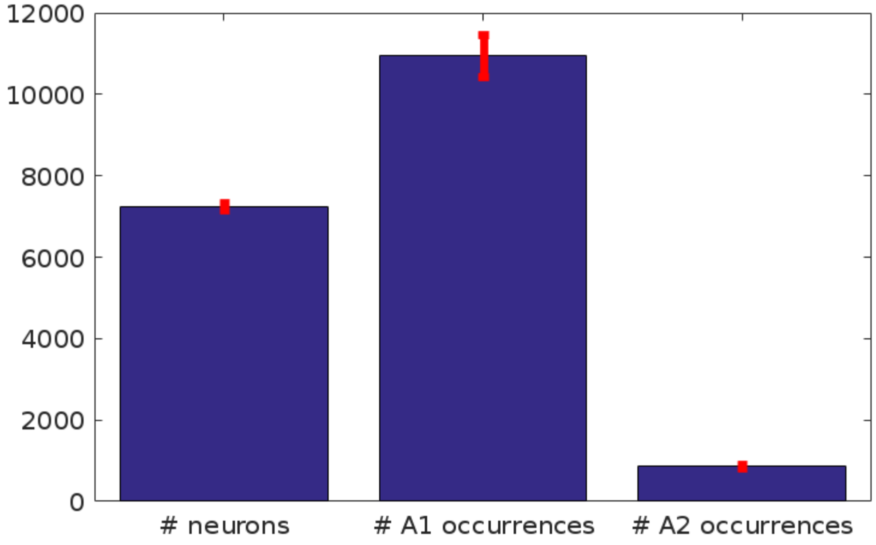

Figure S5: This figure shows the final number of neurons after simulation of layer formation in human temporal cortex (left), the number of cells that have died during development due to A1-type apoptosis (middle), and the number of neurons that have died due to A2-type apoptosis. In accordance with experimental observations, the occurrence of apoptosis during cortical development is of the same order of magnitude as the number of surviving neurons. Due to simplification of preplate splitting process in our model, apoptosis during generation of MZ and layer 6 cells was not included here.

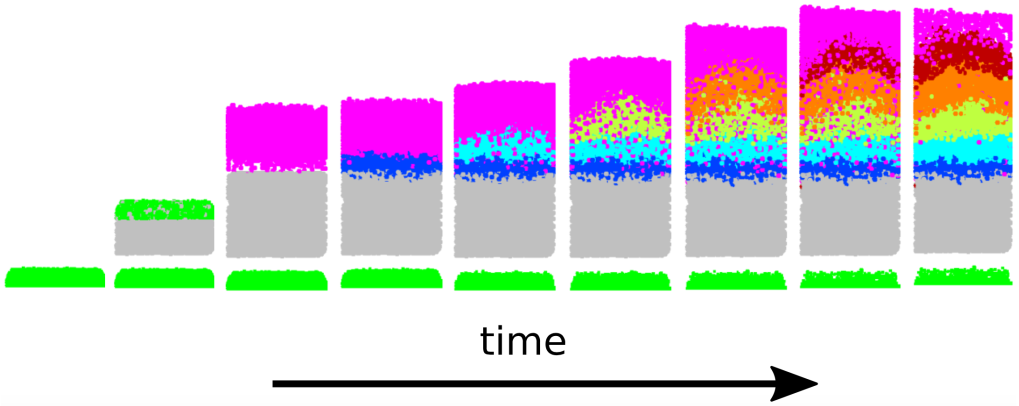

Figure S6: Sequence of layer formation in the extended scenario including subplate cells. Here, the model includes the splitting of the preplate into subplate cells (grey) and marginal zone cells (purple). This is accomplished via the initial external substance gradient that allows cells to differentiate themselves into subplate cells and marginal zone cells, depending on the sensed concentration. To this end, initially migrating cells travel along the radial axis until they sense a concentration that exceeds an initially set threshold of $1 + r \cdot 4$, where r denotes a uniformly distributed variable in the interval $[0, 1]$. After stopping, these cells become subplate cells if their sensed concentration is below a prespecified threshold, or alternatively marginal zone cells. In this way, the subsequently developing layer 6 cells stop migrating when they sense marginal zone cells, hence splitting the preplate into subplate cells and marginal zone cells. This particular simulation yielded 223 layer 1 cells, 3,401 layer 2/3 cells, 1,528 layer 4 cells, 1,519 layer 5 cells and 859 layer 6 cells, in good agreement with measurements from human temporal cortex.
